# Isolation and typing techniques for circulating tumor cells

**DOI:** 10.1186/s40364-026-00901-7

**Published:** 2026-01-28

**Authors:** Shuyan Wu, Shunzi Rong, Anqi He, Yong Xia, Fuyan Xu

**Affiliations:** 1 Joint Lab of Reproductive Medicine of SCU-CUHK, Lab of Reproductive Genetics and Epigenetics, Department of Obstetrics/Gynecology, Key Laboratory of Birth Defects and Related Disease of Women and Children of MOE, West China Second University Hospital, West China School of Medicine, Sichuan University, Chengdu, 610041 China; 2 Rehabilitation Medicine Center, Rehabilitation Medicine Research Institute, Key Laboratory of Rehabilitation Medicine in Sichuan Province, West China Hospital, West China School of Medicine, Sichuan University, Chengdu, 610041 China; 3 West China School of Pharmacy, Sichuan University, Chengdu, China

**Keywords:** Circulating tumor cells, Liquid biopsy, Cell isolation technologies, Phenotypic and molecular typing, Cancer biomarkers

## Abstract

Circulating tumor cells (CTCs) are tumor cells that detach from primary tumors or metastatic sites and enter the peripheral blood. As an important biomarker for liquid biopsy, their detection and analysis have offered a promising avenue in early diagnosis, treatment response monitoring, and prognosis evaluation of tumors. Given the extreme rarity and high heterogeneity of CTCs, efficient and accurate separation and typing have become a primary research focus. This article systematically reviews the mainstream technologies for CTC separation and enrichment, including positive/negative selection based on immune markers, size/deformation/charge separation based on physical properties, and emerging microfluidic platforms; Furthermore, we discuss subtyping strategies for CTC phenotypes and genotypes, encompassing the combined application of surface markers such as EpCAM, CK, Vimentin, gene mutation and copy number variation analysis, transcriptome and proteomic feature analysis, etc.; Additionally, we highlight advancements in functional evaluation techniques such as single-cell culture, metabolic labeling, and in vivo tracking which provide insights into the stemness, drug resistance, and metastatic propensity of CTCs. Finally, we explore the prospects of integrating artificial intelligence and multi omics in CTC typing, pointing out that despite rapid technological progress, challenges in standardization, detection sensitivity, and clinical validation remain significant hurdles to their routine translational application. The review aims to provide a technical framework and cutting-edge perspective for CTC research, and promote its application in personalized tumor management.

## Introduction

The high mortality rate of cancer is mainly attributed to the development of distant metastasis, and CTCs, as a key mediator in the process of tumor metastasis, have received increasing attention in recent years. CTCs refer to tumor cells that shed from primary or metastatic tumors and enter the bloodstream. Although their concentration is extremely rare in peripheral blood (e.g., 1–10 cells per 7.5 mL of blood), they carry the genetic and phenotypic information of the tumor and are one of the most representative biomarkers in “liquid biopsy” [1]. Compared with traditional tissue biopsy, CTC detection has the advantages of including minimal invasiveness, dynamic, and real-time reflection of tumor evolution status, offering a complementary strategy for early screening, efficacy evaluation, drug resistance mechanism research, and recurrence monitoring of cancer [2].

Although CTC research dates back to the 19th century, routine clinical feasibility has only been realized in the last two decades, driven by iterative advancements in enrichment and detection technologies [3]. CTC, as a highly heterogeneous population, not only exhibits significant differences among different patients, but also has different phenotypes and molecular characteristics even within the same patient at different time points or locations. The epithelial mesenchymal transition (EMT) process further exacerbates its heterogeneity, posing a “missed detection” problem for traditional capture strategies that rely on epithelial markers such as EpCAM [4]. Consequently, a diversified separation technology ecosystem is emerging, evolving from single-biomarker capture to multi-marker combinations and physical property identification.

**Fig. 1:**
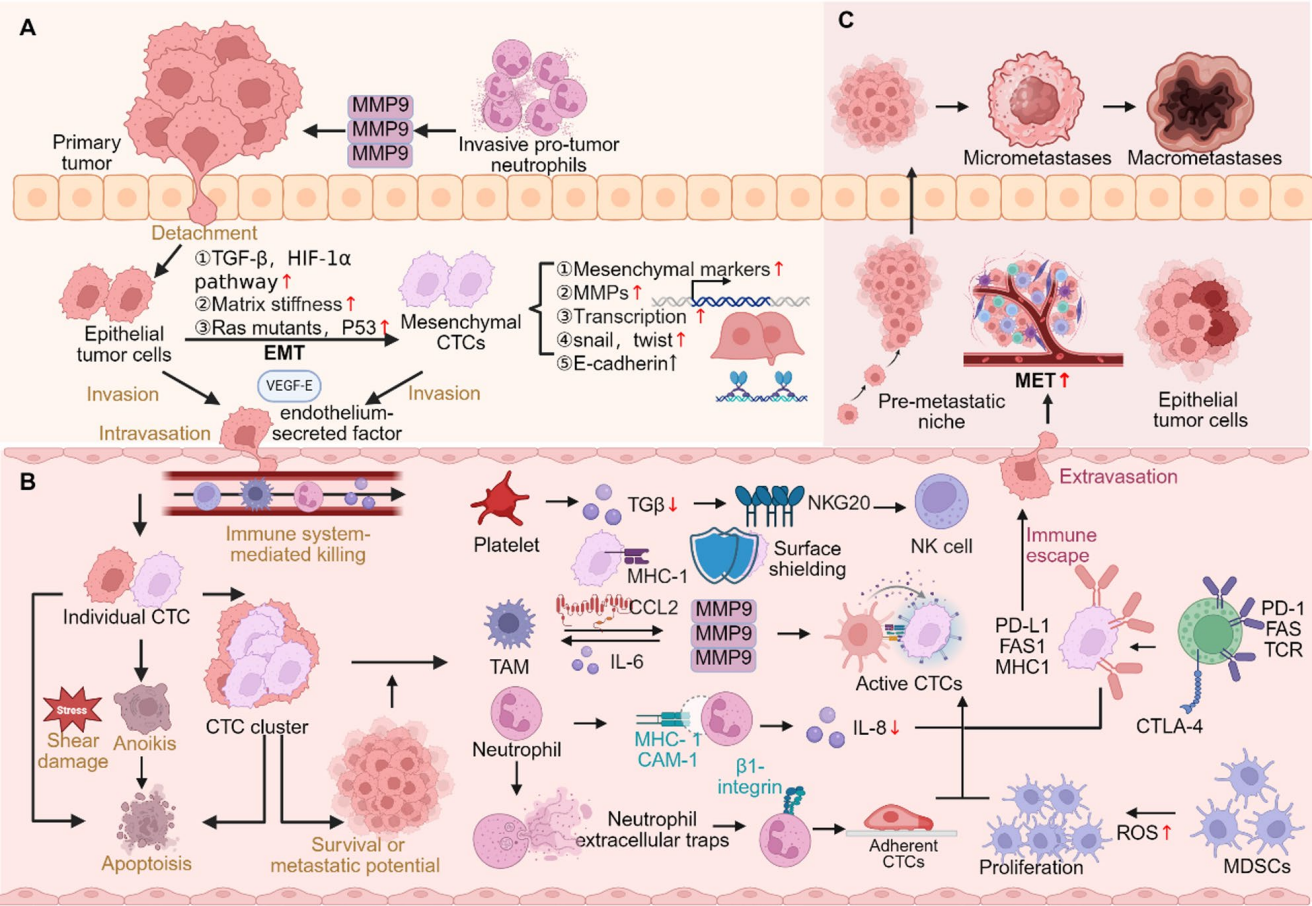
Schematic representation of CTCs during cancer progression and metastasis. (**A**) Within the primary tumor, microenvironmental signals, ECM remodeling, and genetic alterations induce EMT, promoting tumor cell detachment, invasion, and intravasation. (**B**) In circulation, single CTCs face shear stress, anoikis, and immune attack, whereas CTC clusters exhibit higher survival and metastatic potential. Interactions with platelets, neutrophils, Tumor-Associated Macrophages (TAMs), and Myeloid-Derived Suppressor Cells (MDSCs) enhance immune evasion and facilitate dissemination. (**C**) Surviving CTCs extravasate, enter dormancy, and later undergo mesenchymal-epithelial transition (MET) within the pre-metastatic niche, driving micrometastasis formation and macrometastatic growth

At the same time, the functional evaluation of CTC has become a key factor in determining its clinical significance. The development of single-cell analysis technology enables researchers to deeply characterize CTCs, analyze their gene mutations, copy number variations, transcriptome, and proteomic expression characteristics, thereby revealing their potential stemness, chemotherapy resistance, and metastatic ability [5]. In addition, the integration of artificial intelligence, big data analytics, and multi-omics technologies is advancing the development of sophisticated classification systems, promoting CTC from “existence detection” to “functional interpretation“ [6]. Figure 1 illustrates the key pathways of CTC in the occurrence, circulation, and metastasis of cancer, which helps to understand the biological significance and research value of CTC.

However, current CTC research still confronted with multiple unresolved challenges, such as inconsistent sensitivity and specificity of enrichment methods, lack of unified CTC definition standards, and differences in applicability between different cancer types. In addition, how to effectively integrate the results of separation and typing and guide personalized clinical treatment is also a key direction that future CTC research needs to focus on. This review begins by examining the technology behind CTC separation and will systematically review the progress made in their classification and functional assessment, aiming to provide a overview that acknowledges both the potential and the current limitations of CTCs, serving as a comprehensive resource for both foundational research and clinical translation of CTCs.

## CTC isolation and enrichment techniques

**Fig. 2:**
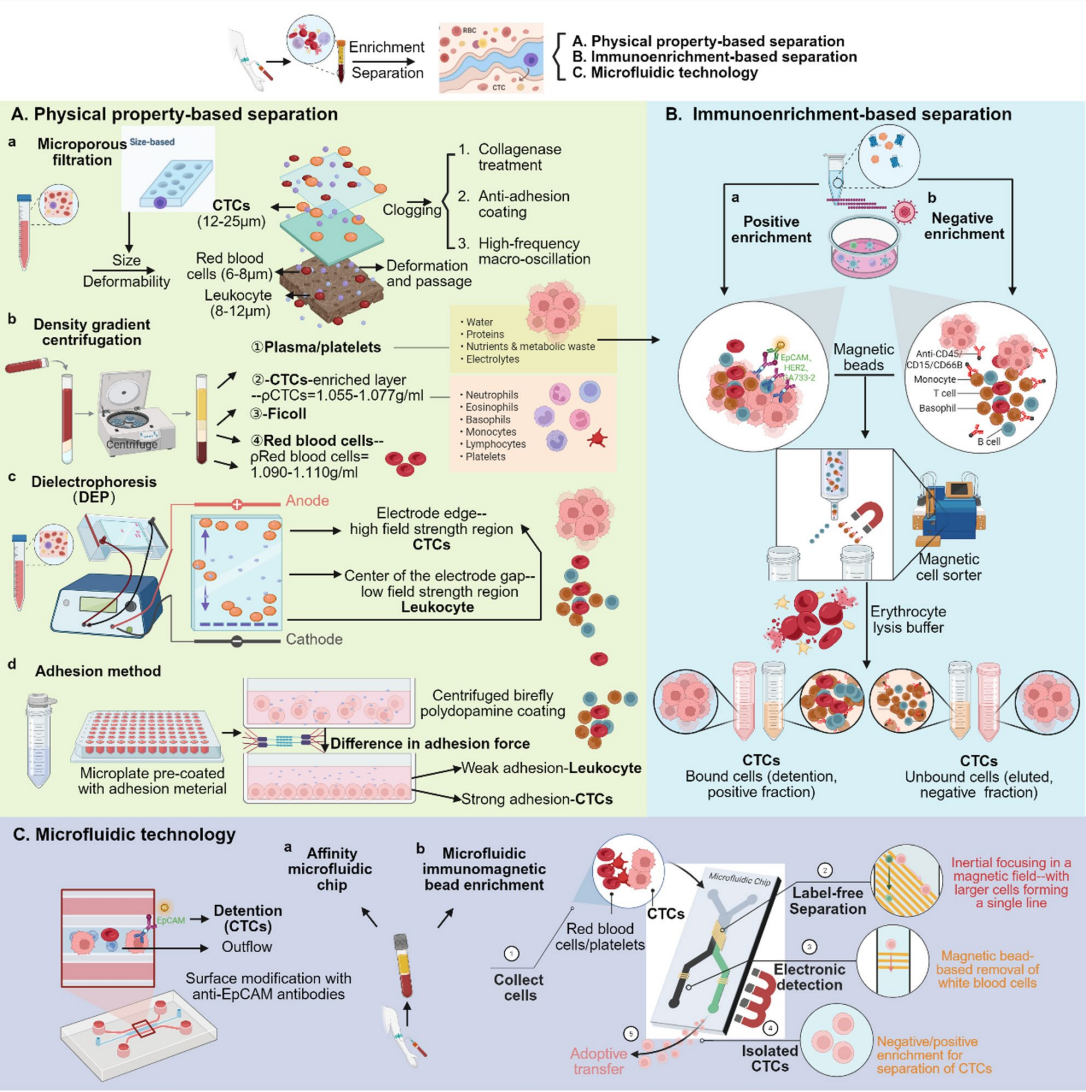
Major strategies for CTC isolation and enrichment. (**A**) Physical property-based separation: (a) Microporous filtration — based on size and deformability; (b) Density gradient centrifugation — based on cell buoyant density; (c) DEP — exploits differences in dielectric properties; (d) Adhesion capture — utilizes stronger adhesion of CTCs to coated surfaces. (**B**) Immunological enrichment: Positive selection via tumor-specific antigens (e.g., EpCAM) or negative depletion of leukocytes (e.g., CD45). (**C**) Microfluidic technology: Combines affinity-based microchips and immunomagnetic bead enrichment for high-efficiency CTC capture

Given the well-documented role of CTCs in tumor progression, the efficient and accurate isolation is a prerequisite for their clinical translation. This section provides a systematic overview of the current mainstream CTC isolation and enrichment technologies, covering surface marker identification to physical property screening to advanced microfluidic systems. As shown in Fig. 2, the current CTC separation technologies are mainly divided into three categories: immuno-enrichment, physically based separation, and microfluidic platform, each with its own distinct advantages and inherent limitations.

### Immunoenrichment-based separation methods

The most widely used technique for CTC isolation is immunoenrichment-based isolation, which is a method of isolation and enrichment using antibodies that selectively bind to cell surface antigens based on the fact that specific tumor cells express cell surface markers that are different from those of blood cells.

#### Positive selection

Positive selection is one of the most widely used strategies for CTC isolation. The basic principle is to utilize the highly specific binding of tumor cell surface-specific antigens (e.g., EpCAM) to the corresponding antibodies to capture CTCs directly from the bloodstream by means of immunomagnetism or interfacial functionalization for their enrichment and detection. Epithelial Cell Adhesion Molecule (EpCAM) is one of the most commonly used capture targets, which is widely expressed on CTCs of various tumors of epithelial origin, but hardly expressed in normal leukocytes. EpCAM-positive cells are rarely detected in the peripheral blood of healthy populations, making targeting EpCAM a frequent target of enrichment CTCs [7, 8].

The CellSearch system, the first CTC detection platform approved by the U.S. Food and Drug Administration (FDA), performs positive selection based on EpCAM. The system utilizes magnetic nanoparticles coated with antiEpCAM antibody for immunomagnetic enrichment of CTCs and fluorescent labeling to detect specifically expressed cellular phenotypes for automated identification [9]. CellSearch is well standardized and clinically reproducible, and has been widely used for treatment evaluation and efficacy monitoring of a variety of metastatic tumors, such as metastatic prostate, ovarian, and breast cancers [10]. However, this system is highly dependent on EpCAM expression and has limited sensitivity in CTCs with low expression levels. To overcome the problems of low CellSearch release efficiency and impaired cellular function, the MagSweeper system was developed and applied for the gentle capture of rare CTCs. It removes cells that do not bind magnetic beads through a magnetic rod in a low-shear environment while maintaining the integrity and function of the CTCs, providing better samples for subsequent transcriptomic and functional experiments [11].

Another commercialized platform, AdnaTest, is also based on EpCAM’s positive selection. The system works by encapsulating a combination of multiple antibodies. (e.g., EpCAM, HER2, GA733-2) of immunomagnetic beads for CTC enrichment and combined with multiplex RT-PCR for detection of relevant transcripts (e.g., HER2, MUC1), which enhances the efficiency of the detection of CTCs in breast, prostate, ovarian and colon cancers [12]. Although AdnaTest is superior to CellSearch in terms of detection sensitivity, it requires a higher blood sample volume, often requiring multiple tubes of blood, which increases operational complexity [13]. For example, in metastatic Castration-Resistant Prostate Cancer (mCRPC), CellSearch has relatively limited detection sensitivity, whereas AdnaTest and mRNA-based ddPCR methods show higher sensitivity [13]. A major limitation of EpCAM-dependent methods is tumor heterogeneity and phenotypic loss due to EMT. During EMT, CTCs may down-regulate or lose the expression of epithelial markers (e.g., EpCAM, CK), and instead express mesenchymal phenotypes such as Vimentin or N-cadherin, which have higher metastatic potential but are difficult to be recognized by traditional positive selection methods due to the lack of EpCAM expression, and thus pose a risk of false negatives [14]. In order to improve the efficiency of positive selection in capturing CTCs of different phenotypes, multi-target combination strategies have emerged. For example, combining EpCAM with EGFR antibody can significantly increase the detection rate of CTCs in NSCLC patients, and the capture efficiency is significantly improved [15]. In addition, the combination of targeting specific molecules in the EMT process, such as CD44, PD-L1, and c-MET, can also be used to capture a subpopulation of CTCs with high metastatic potential [16].

In recent years, researchers have also developed EpCAM non-dependent positive selection techniques. Nanoprobes based on negative charge changes on the cell membrane surface can target the charge characteristics induced by glycolysis in tumor cells to enrich CTCs with a detection sensitivity of up to 81.6% [5]. A new method called BloodFlow, which immunomagnetically enriches CD138 + circulating plasma cells and combines it with next-generation flow cytometry (NGF), enables highly sensitive detection of very low levels of residual lesions in the peripheral blood of patients with multiple myeloma, which shows high predictive value, especially during maintenance therapy, and can be effective in predicting progression-free survival [17]. In addition, novel functionalized interfaces have dramatically expanded the means of positive capture. For example, photo-functionalized TiO₂ membranes can immobilize EpCAM antibodies by electrostatic adsorption for efficient separation of hepatocellular carcinoma CTCs [18]. Bionic peptidemodified interfaces (e.g., LAPTM4B targeting peptide) can recognize both epithelial and mesenchymal phenotypes and achieve high capture rates in liver cancer patients [19].

However, even with a multi-target strategy, dynamic expression changes of CTC markers remain one of the intractable challenges of positive selection. Single-cell sequencing studies have shown that EpCAM expression varies greatly in CTCs from the same patient [20], suggesting that CTC detection needs to be integrated with multi-omics analysis tools to avoid missed detection due to insufficient expression of a single target [21].

In conclusion, as the core technology of CTC enrichment, positive selection still needs to optimize its target combination and enrichment mechanism to adapt to the complex reality of CTC heterogeneity and phenotypic diversity.

#### Negative selection

In contrast to positive selection, negative selection based on nonspecific enrichment by leukocyte removal indirectly enriches CTCs by removing a high abundance of leukocytes (CD45⁺) and erythrocytes from the blood, which is particularly suitable for the detection of CTCs with low EpCAM expression or mesenchymal phenotype [22]. The core strategy is to remove leukocytes and retain unlabeled CTCs using immunomagnetic beads or microfluidic devices with anti-CD45, CD66b or CD15 antibodies.

For example, the PowerMag system utilizes CD45 magnetic beads to negatively enrich and bind to membrane-bound protein V to remove apoptotic cells to improve CTC purity [23]. CTCs with EMT and cancer stem cell phenotypes from breast cancer patients were successfully captured by negative selection using the ApoStream^®^ platform [24]. As well as size-based negative selection was successfully used for CTC enrichment and detection of specific mutations in lung cancer patients [25].

However, the main limitations of negative selection are the low purity of the resulting product and the fact that residual leukocytes interfere with downstream analysis. The presence of CD45-negative cells in the blood of healthy donors suggests that a combination of morphological or molecular markers is required to exclude false positives [26]. Enrichment products contain high levels of non-target cellular debris that need to be combined with a secondary screen to improve specificity, and leukocyte contamination remains a significant problem even in CD45/CD66b dual-target negatively enriched CTC samples [27].

Multi-marker combined negative screening improves on these limitations. Immunomagnetic negative enrichment combined with light-induced dielectrophoresis was used to further isolate CTCs from the blood of leukemia patients to improve the purity of the isolation [28]. Flow cytometry coupled with immunomagnetic beads (e.g., the FETCH system) reduces nonspecific adsorption by adjusting magnetic field strength and buffer ion concentration [29]. In addition, size-based composite enrichment methods (e.g., ISET technology) enhance the capture sensitivity of free tumor cells from gastric cancer peritoneal lavage fluid [30]. Future directions include the development of high-throughput integrated devices, such as 3D-printed monolithic devices [31], and the optimization of CTC recognition algorithms in conjunction with artificial intelligence [32].

### Separation methods based on physical properties

Although immunoenrichment methods are widely used due to their high specificity, their antigen-expression-dependent nature also limits their application in heterogeneous CTCs. To compensate for such limitations, researchers have proposed a variety of separation techniques based on physical properties of cells for label-free, high-throughput CTC enrichment.

#### Size and deformability: size filtration method

Physical separation of CTCs is largely dependent on the dual properties of their size and deformability. The size of CTCs is usually 12–25 μm, which is significantly larger than that of red blood cells (6–8 μm) and platelets (2–4 μm), and partially overlaps with leukocytes (8–20 μm) [33]. Size-based separation methods utilize the size difference between CTCs and blood cells for efficient enrichment through physical sieving or inertial focusing techniques. For example, the MetaCell^®^ system uses polycarbonate microfiltration membranes (8 μm pore size) to directly filter whole blood samples, allowing for rapid capture of CTCs while preserving their activity for subsequent culture [34]. Compared to more rigid leukocytes, CTCs exhibit higher deformability due to cytoskeletal variability, a property that can be efficiently screened by microfluidic channel design. Spiral microfluidic channels separate large-sized CTCs from blood cells through the inertial focusing effect, and specifically designed chips utilize the synergistic effect of inertial lift and Dean traction to achieve the separation of CTCs from leukocytes. These chips are designed to induce cell deformation through the contraction-expansion channel to take advantage of the difference in rigidity for screening [35]. A newly developed high-throughput microfluidic device could successfully enrich a large number of CTCs from leukocyte separation products by magnetic lensing and inertial focusing, and further revealed the heterogeneity of CTCs by single-cell sequencing. Another study enhanced the throughput and separation efficiency by combining inertial focusing and other techniques [36]. Sequential filtration allowed the separation of CTC single cells from clusters, which were found to correlate with specific metastatic risks [37].

However, the generalizability of this method to heterogeneous CTCs still requires large-scale clinical validation. The main challenges currently faced include the lack of purity due to leukocyte residues and the possible missed detection of small-sized CTCs or CTCs undergoing EMT [38]. For example, the FAST system has a low capture rate for small-sized CTCs, and the combination of label-free technologies (e.g., AccuCyte^®^) can significantly improve the capture efficiency [39]. In the future, it is expected that microchannel geometries (e.g., trapezoidal cross-section design) can be optimized to increase the separation rate, combining deformability and surface charge for multi-dimensional screening [40], or integrating artificial intelligence to regulate separation conditions in real time [41], new breakthroughs are expected to be realized.

#### Density: density gradient centrifugation

Density gradient centrifugation achieves separation based on the density difference between CTC (1.055–1.077 g/mL) and erythrocytes (1.090–1.110 g/mL) and leukocytes (1.065–1.092 g/mL). The conventional Ficoll method often suffers from severe monocyte contamination, leading to its gradual replacement by improved density gradient systems like OncoQuick^®^, which utilizes a porous barrier to enhance CTC purity [42]. For example, density gradient combined with negative enrichment of CD45 immunomagnetic beads can be used to isolate heterogeneous CTCs with a high survival rate from the blood of oral squamous carcinoma patients and reveal their drug-resistance-related gene expression profiles by single-cell transcriptome analysis [43]. In clinical practice, the density gradient method is often used as a pretreatment step coupled with microfluidic or immunocapture techniques, for example, utilizing poly(ethylene oxide) concentration gradients coupled with inertial microfluidics to achieve better diagnostic accuracy [44].

Despite its ease of operation and low cost, the density gradient method has significant limitations. First, the separation efficiency is affected by cell morphology and medium stability, such that CTC aggregates are lost due to abnormal settling velocity [45]; second, it is difficult to distinguish CTCs from Mesenchymal Stem Cells (MSCs) (density 1.030–1.060 g/mL). Improvement strategies include the development of gradient-continuous media (e.g., Percoll-Iodixanol composite system) and automated centrifugation devices (e.g., CTC-iChip integrated centrifugation with inertial focusing from Jounce), the latter of which can shorten processing time [46]. In addition, gold nanoparticles modifying CTC to amplify the density difference [47] and microgravity simulations of space experiments revealing the settling kinetics of CTC in ultra-low gravity [45] are expected to provide new ideas to overcome the limitation.

#### Dielectricity: dielectrophoresis techniques

Dielectrophoresis (DEP) technology induces cell polarization through a non-uniform electric field and exploits the differences in dielectric properties (e.g., membrane capacitance, cytoplasmic conductivity) between CTCs and blood cells to achieve label-free separation. For example, counting bipolar electrode arrays at 50 kHz can cause specific tumor cells and monocytes to migrate in opposite directions, enabling efficient capture and maintaining cell viability [48]. Similarly, the chip electrode layout was optimized by COMSOL simulation to achieve CTC, leukocyte, and platelet separation with reduced power consumption at ± 2 V [49].

The core challenge of the DEP technique lies in the dielectric heterogeneity of cell populations. For instance, while CTCs and leukocytes typically exhibit distinct crossover frequencies, phenotypic variations (e.g., EMT) or heterogeneity in tumor cells can alter their dielectric parameters (such as membrane capacitance), potentially leading to overlap with blood cells and fluctuating capture efficiency [50]. Solutions include multi-frequency alternating electric fields [51], real-time impedance feedback systems to monitor cellular dielectric properties in real time and adjust the frequency [52], and 3D electrode arrays and microdroplet encapsulation technologies to enhance processing capacity [53].

#### Adhesion: functionalized surface capture

Adhesion-based separation techniques achieve capture by modulating the physical interactions (e.g., hydrophobicity, surface charge) of CTCs with the material surface without relying on antibodies. For example, the hydrophobicity of CTC membrane lipid bilayers (contact angle 70°-85°) is significantly higher than that of leukocytes (50°-60°); and the surface charge of CTCs (-15 to -25 mV) binds to positively charged polydopamine coatings. And the bovine serum albumin interface of cancer cell membrane-coated microarrays was utilized to capture CTCs by topology matching and then release them non-destructively with complementary DNA strands [54]. A nanoporous micropillar array chip enabled spatially resolved immunocapture of CTCs by modulating the fluid velocity and surface topology, which significantly enhanced the capture efficiency of tumor cells with low expression of EpCAM and successfully differentiated stage from advanced cases in breast cancer patients [55]. Similarly, specifically coated microtiter plates were developed to rapidly isolate CTCs through differences in adhesion force at a significantly lower cost than the antibody method [42]. Temperature-sensitive hydrogels (PNIPAM) act as a dynamic interface to release CTCs at 37 °C, avoiding enzymatic damage [47].

However, nonspecific adsorption of fibronectin or apoptotic cells can interfere, while throughput is limited and batch mode is difficult to adapt to large samples [54]. Current directions for improvement include the application of bionic coatings to enhance capture stability and the use of laser-engraved microwell arrays to enhance single-cell capture efficiency [56].

### Microfluidic technology

With the development of nanotechnology and bioengineering, microfluidic platforms have gradually become an important tool in CTC isolation studies. Compared with traditional immunological and physical methods, they have significant advantages in terms of throughput, automation and cell activity maintenance. Its latest research progress and representative systems are described below.

Microfluidic systems precisely manipulate fluids through micrometer-scale channels and structures, utilizing hydrodynamic properties at the micro-scale(e.g., laminar flow, inertial focusing, acoustic force, magnetic force, etc.) for efficient processing of biological samples [57]. Microfluidics continues to innovate in the field of CTC separation with a focus on improving throughput, purity and downstream compatibility. Devices relying on simple filtration or affinity capture often face low recovery rates and leukocyte contamination. Current research has shifted towards combining multiple separation mechanisms (size fractionation, inertial focusing, dielectrophoresis) to improve efficiency and specificity [58]. Notable advances include the integration of immunomagnetic techniques into microfluidic platforms, the most common technique utilizing antibodies that can be coupled to magnetic nanoparticles or immobilized on the wall of the microfluidic chip, which in turn captures CTCs by immunoaffinity Capturing CTCs from whole blood.

Physical property-based techniques primarily exploit size differences and achieve separation by designing specific microchannel structures (e.g., spiral channels, barrier arrays). For example, a two-stage integrated chip combines inertial focusing and dielectrophoresis to achieve high separation efficiency and throughput [36]. The advantage of such techniques is that they do not require labeling and are suitable for heterogeneous CTCs, but they may miss small CTCs or mistakenly trap large leukocytes, and high flow rates may lead to mechanical damage to cells [59]. Acoustic microfluidics utilizes acoustic force fields for contactless sorting, such as acoustic enrichment systems that support high recovery rates and CTC cluster detection [20]. The advantage is high cellular activity, but its low throughput, high equipment complexity, and demanding in-channel flow field stability limit clinical large-scale applications [57].

Bioaffinity-based capture technologies use antibody-functionalized microarrays (e.g., EpCAM, EGFR antibodies). Antibody-functionalized microfluidic chip (AFM Chip) has achieved efficient capture and multi-cancer application in breast cancer [60]. However, such methods rely on CTCs’ surface marker expression and may miss detection of CTCs undergoing EMT [61]. Multifunctional probe integration is another effective method, such as antibody-aptamer hybridization probe (Apt-mAb) combined with microfluidics to achieve universal capture and sensitivity enhancement and reusability [62]. Similarly, to overcome the limitations of a single technology, hybrid systems combining physical screening with biocapture can enhance performance. For example, i-Mag chips were purified by inertial screening and magnetic bead labeling of leukocytes for higher CTC purity [63]. The introduction of folate-functionalized magnetic nanoparticles achieved high capture efficiency, activity, and downstream compatibility, but the hybrid system design is complex and requires multi-step operational integration, which may lead to the loss of rare CTCs [64].

Microfluidic systems offer several advantages over traditional CTC isolation methods, but the complexity of device fabrication, the need for specialized equipment, and the heterogeneity of CTCs pose a challenge for widespread and consistent application. Most studies rely on limited sample validation and lack large-scale clinical data support. Whereas PANDA chip is a label-free microfluidic sorting system capable of efficiently capturing CTC clusters in undiluted blood with high recovery of blood components, this technology offers unprecedented throughput and sensitivity for CTC cluster research [65]. In the future, there is a need to balance sensitivity with specificity, throughput with activity, and to integrate isolation, detection, and analysis into a single workflow. The utility and reliability of the system can be further enhanced through the integration of multimodal technologies (e.g., acoustic-magnetic synergistic sorting), the integration of single-cell analysis (e.g., in situ gene sequencing), and the establishment of standardized processes [66]. And portable device development (e.g., based on paper-based or flexible materials, such as cotton fiber matrices by Arora et al.) is expected to drive bedside screening [67]. Integration of artificial intelligence and machine learning algorithms can improve the accuracy and efficiency of CTC detection, paving the way for personalized cancer treatment [68].

### Market-available CTC isolation platforms

While numerous isolation technologies remain in the laboratory development phase, the rapid growth of the liquid biopsy industry has propelled several platforms into commercial availability. These systems are pivotal for bridging the gap between benchtop innovation and clinical utility, offering standardized workflows for multicenter trials. Currently, commercial platforms can be broadly categorized based on their isolation principles: immunoaffinity-based systems and physical property-based systems.

#### Immunoaffinity-based platforms

The CellSearch system (Menarini Silicon Biosystems) remains a widely recognized benchmark for CTC verification. Relying on ferrofluid nanoparticles coated with anti-EpCAM antibodies, it is the first FDA-cleared actionable assay for detecting CTCs [9]. However, its reliance on EpCAM limits its utility in detecting varying phenotypes, particularly those undergoing EMT. To address this, AdnaTest (Qiagen) combines immunomagnetic enrichment (targeting EpCAM and HER2) with multiplex RT-PCR detection. Unlike CellSearch which enumerates cells, AdnaTest focuses on molecular characterization and has demonstrated higher sensitivity in specific contexts, such as metastatic castration-resistant prostate cancer (mCRPC) [13]. Furthermore, for downstream single-cell analysis, the DEPArray™ system (Menarini Silicon Biosystems) utilizes dielectrophoresis to manipulate individual cells. Although primarily a sorting rather than enrichment tool, it is frequently paired with CellSearch to isolate pure single CTCs for whole-exome sequencing, revealing mechanisms of drug resistance such as MYC amplification in SCLC [69].

#### Physical property-based platforms

Recognizing the limitations of marker-dependent capture, platforms exploiting cell size and deformability have gained prominence. Parsortix (Angle plc) employs a microfluidic stepped cassette to capture cells based on size and deformability, allowing for the viable capture of mesenchymal CTCs for downstream analysis like transcriptome profiling [70]. ISET (Rarecells Diagnostics) [71] utilizes isolation by size of tumor cells through filtration. It has demonstrated superior sensitivity in early-stage cancers, such as achieving an 80% detection rate in early NSCLC patients compared to lower rates with EpCAM-based methods [72]. Other notable platforms include ScreenCell [73], which offers filtration-based devices optimized for cytomorphological analysis, and Genesis (Bio-Rad Laboratories) [74, 75], which also focuses on physical isolation.

#### Integrated and emerging commercial solutions

Recent commercial developments aim to integrate multiple separation mechanisms to enhance purity. The TellDx CTC platform (TellBio, Inc.) [76, 77] incorporates the CTC-iChip technology, which combines negative depletion of leukocytes with inertial focusing [78]. This strategy effectively removes blood components while retaining untagged tumor cells, achieving high-purity isolation suitable for molecular profiling. Additionally, CD-PRIME system (Clinomics Inc.) [79, 80] offers label-free isolation based on fluid dynamics.

These commercialized systems are crucial for standardizing CTC isolation across different medical centers. However, discrepancies in sensitivity and the definition of “CTC positive” between platforms (e.g., CellSearch vs. ISET) highlight the ongoing need for cross-platform validation to facilitate large-scale clinical application.

### Emerging separation technologies

While commercial platforms rely on established principles, the frontier of research is continuously expanding. Microfluidics has significantly improved the throughput and efficiency of CTC separation and supported preliminary downstream analysis through its powerful integration capabilities and versatility. However, in order to more deeply analyze the molecular heterogeneity and dynamic functions of CTCs and their precise roles in metastatic cascades, researchers are continuously exploring and developing more groundbreaking and emerging separation technologies, aiming to achieve precise manipulation, nondestructive capture, and real-time functional assessment at the single-cell level.

#### Nanomaterials applications

Nanomaterials show great potential in CTC separation due to their unique surface, small size and quantum effects, and their high specific surface area and functionalizable properties enable efficient specific capture. A variety of nanomaterials, including magnetic nanoparticles, gold nanoparticles, quantum dots, and metal-organic skeletons (MOFs) can be used for CTC separation and enrichment [81]. For example, tannic acid-functionalized magnetic nanoparticles (MNPs-TA) achieve efficient capture through the specific interaction of polyphenolic structures with the glycocalyx on the surface of cancer cells and significantly inhibit the nonspecific adsorption of peripheral blood mononuclear cells [82]. Bionic immunomagnetic gold hybrid nanoparticles (CM-Fe3O4@Au-Ab) effectively reduced the non-specific binding to leukocytes by leukocyte membrane camouflage technology, and combined with inductively coupled plasma mass spectrometry (ICP-MS) detection, the CMFe3O4@Au-Ab were highly sensitive in mimicking clinical blood samples [83]. Synthesized through antibody- and N-cadherin-containing nanoparticles, these MOF-based double-antibody nanoparticles overcame the heterogeneity of CTCs caused by EMT through the synergistic effect of antibodies EpCAM and N-cadherin, resulting in an improved efficiency of CTC capture in blood from gastrointestinal tumor patients [84]. The light-induced hydrogel response platform integrates gelatin nanoparticles (Gnps) and chondroitin sulfate methacryloyl (CSMA), which are selectively cured to encapsulate the target CTCs by a 405 nm laser to achieve controlled release and good cellular activity [85].

However, nanomaterials still face challenges in practical applications, such as the lack of standardized preparation processes, uncertainty of long-term biosafety, protein corona formation on the surface of nanomaterials that may mask the targeted ligands and reduce the capture efficiency, and insufficient stability in complex biological samples [86]. In the future, multifunctional integrated nanoplatforms (e.g., diagnostic and therapeutic integrated systems with both capture, imaging, and therapeutic functions) can be further developed, as well as high-throughput, automated manipulation of nanomaterials through microfluidics [64]. Surface modification strategies for multifunctional nanomaterials are also being innovated, such as polydopamine coatings for simultaneous solidification of multiple ligands, and DNA tetrahedral structures for fixation, while the DNA tetrahedral structure can precisely control the spatial arrangement of ligands and significantly enhance the capture efficiency [87]. The excellent photothermal conversion properties and rich surface chemistry of novel nanomaterials, such as black phosphorus nanosheets and MXene, offer new possibilities for the efficient capture and controlled release of CTCs [88]. Whereas the surface properties of nanomaterials (e.g., charge, hydrophobicity, etc.) have a significant effect on the capture performance of CTCs, smart-responsive material openings of nanocarriers responsive to multiple stimuli (e.g., light, pH, enzyme) can be developed, which can enable the dynamic capture and controllable release of CTCs [89].

#### Nucleic acid aptamer capture

Nucleic acid aptamers, as single-stranded DNA or RNA molecules screened by SELEX technology, are capable of binding target molecules with high specificity and affinity. Compared with traditional antibodies, aptamers have the advantages of small molecular weight, easy chemical modification, good stability, and low immunogenicity, which make them especially suitable tools in oncology, including applications for capturing heterogeneous CTCs [90]. For example, dual-targeted multivalent aptamers(recognizing both EpCAM and HER2) achieved efficient capture of heterogeneous CTCs through a three-dimensional DNA walker mechanism (3D DNA walker) with significantly higher sensitivity than conventional single aptamers [91]. A novel peptide targeting N-cadherin-Functionalized magnetic nanoparticles targeting N-cadherin can effectively capture mesenchymal CTCs and distinguish subpopulations, which can solve the problem of underdetection of EpCAM-negative CTCs [92]. DNAzyme -functionalized proteins biotinylated strategy (DPPB) reduces the subpopulation capture bias and improves the performance of clinical samples by labeling biotin on the surface of CTCs in situ [93]. The introduction of tetrahedral framework nucleic acid technology significantly enhances the stability of aptamers, and its rigid structure can precisely control the spatial arrangement of binding sites and improve the capture efficiency [94]. The assembly of multivalent aptamers (TEA(n)) using tetrahedral DNA frameworks (TDFs) significantly enhanced binding stability in the serum environment, with a significant increase in dissociation constants (Kd) over conventional aptamers, and achieved a high classification accuracy of treatment response in hepatocellular carcinoma patient samples [95].

For clinical applications, the advantages of low cost and large-scale synthesis of aptamers have led to significant commercialization potential, but challenges such as the susceptibility to degradation by nuclease in serum and affinity to complex biological environments are still being faced [96]. Chemically modified aptamers (e.g., phosphorothioate modification) have been developed to improve their stability, while multivalent aptamers have been designed to enhance binding capacity through synergistic interactions [92]. In addition, the integration of aptamers with other technologies has shown promising prospects, such as aptamer-functionalized microfluidic chips to achieve high-purity separation of CTCs, and aptamer-mediated magnetic separation to greatly simplify the operation process [97]. Future directions include the development of activatable aptamers, multifunctional aptamers, and smart-responsive aptamers (e.g., light-controlled or pH-responsive), and the establishment of aptamer databases and the application of machine learning algorithms, as well as the improvement of the standardized evaluation system, will accelerate their development and clinical translation [94].

#### Advanced functional magnetic nanomaterials

Magnetic nanoparticle technology has become one of the mainstream methods for the separation and enrichment of CTCs due to its ease of operation, rapid separation and high efficiency. Immunomagnetic separation is achieved through surface functionalization (e.g., antibody, aptamer modification), which combines high capture efficiency with ease of operation. Its core advantage is that it can be rapidly enriched by an external magnetic field, making it suitable for the processing of large-volume blood samples. The synergistic effect of the superparamagnetic iron oxide core and the peripheral functionalized coating endows these materials with unique properties [86].

The new generation of magnetic nanoparticles enhances the performance through innovative design, and the dual stealth design of polyethylene glycol (PEG) spacer arms and protein corona pre-coating (IP-CMNs) significantly reduces non-specific protein adsorption and improves clinical detection rate [47]. High expression of folate receptor on the surface of tumor cells, folate-functionalized Fe3O4 nanoparticles in microfluidic microarrays can be efficiently captured on breast cancer cells (MCF-7) [64]. Hybrid engineered cell membrane-camouflaged magnetic nanoparticles (HE-CM-MNs) efficiently capture heterogeneous CTCs of multiple cancer types by integrating three targets, namely EpCAM, EGFR and Her2, to support downstream mutation analysis [98]. Notably, the particle size and surface charge of magnetic nanoparticles have an important impact on the capture efficiency, and the performance can be significantly improved by optimizing these parameters [86].

However, there are some limitations of this technique, such as the steric hindrance of beads may affect downstream analysis, rare subpopulations (e.g., CTC clusters) are easily lost, and the high concentration of MNPs may trigger the particle aggregation, which affects the capture uniformity [99, 100]. In contrast, the gradient magnetic field design reduces cell damage, while the combination of a microfluidic chip and magnetic separation improves the recovery of rare subpopulations [98]. In the future, the development of multifunctional magnetic nanoparticles (e.g., with combined separation, imaging, and therapeutic functions), intelligent responsive materials (e.g., photo- or magnetically-controlled release), and portable detection devices [101]. The establishment of standardized production processes and large-scale clinical validation will accelerate the translational application of magnetic nanoparticle technology, while the integration with other technologies (e.g., microfluidics, mass spectrometry, etc.) is expected to surmount the obstacle of existing technologies [86].

A variety of current CTC separation strategies have their own characteristics in terms of principle, advantages, and disadvantages, and applicable scenarios, as shown in Table 1.

**Table 1:** CTC isolation and enrichment techniques: principles, strengths and limitations

Methodology		Principles	Strengths	Limitations	References
Immunoenrichment	Positive selection	Capture of CTCs using tumor cell surface antigens (e.g., EpCAM) in combination with antibody specificity	High specificity; high standardization (e.g., CellSearch system); supports multi-target combination strategy to enhance sensitivity	Dependent on antigen expression, easy to miss EMT-type CTCs; dynamic expression changes lead to unstable capture; some platforms are complicated to operate (e.g., AdnaTest requires large blood volumes (multiple tubes))	[4, 13, 15, 102, 103]
	Negative selection	Indirect enrichment of unlabeled CTCs by removing leukocytes (CD45⁺, etc.)	Suitable for CTCs with low EpCAM expression or mesenchymal phenotypes; avoids antigen-dependence issues.	Low product purity (residual leukocyte interference); secondary screening required to exclude false positives; high non-target cell debris	[22, 26, 27]
Physical characterization	Size-based filtration	Utilizes differences in CTC size (12–25 μm) compared to blood cells	Label-free and universal; preserves cellular activity (e.g. MetaCell^®^); CTC clusters can be separated	Small CTC size or EMT type easily missed; leukocyte residue leads to lack of purity; high flow rates may damage cells.	[37, 38, 59, 104]
	Density gradient centrifugation	Separation of CTCs based on differences in density of CTCs and blood cells	Easy and inexpensive to perform; often used as a pretreatment in conjunction with other techniques	Easy to lose CTC aggregates; difficult to differentiate MSCs; separation efficiency affected by media stability	[44, 45]
	Dielectrophoresis	Separation in a non-uniform electric field utilizing differences in dielectric properties (membrane capacitance, conductivity) between CTCs and blood cells	No labeling, cell activity maintained; low power consumption (optimized electrode layout)	Dynamic changes in dielectric parameters affect efficiency (e.g., EMT reduces membrane capacitance); it requires a multifrequency electric field or feedback system optimization.	[49–51]
	Adhesion capture	Physical adsorption using CTC hydrophobicity/surface charge with functionalized materials (polydopamine coatings, etc.)	No antibodies required, low cost; non-destructive release from temperature-sensitive hydrogels (e.g., PNIPAM)	Non-specific adsorption interference (fibronectin/apoptotic cells); limited throughput, difficult to fit large samples	[47, 54]
Microfluidics	Integrated microfluidic chips	Integration of hydrodynamic properties (laminar flow, inertial focusing, etc.) with physical/biological capture mechanisms	Precise manipulation and automation; high cell activity and downstream compatibility; support for multi-mechanism fusion (e.g., iMag chip)	Complex equipment requires specialized instrumentation; low consistency of heterogeneous CTC capture; insufficient clinical large-scale validation	[57, 63]
Emerging technologies	Nanomaterials applications	Utilizing nanomaterials (magnetic particles, MOF, etc.) with high specific surface area and functionalizable properties to specifically capture CTCs	High capture efficiency (e.g., MNPsTA inhibits nonspecific adsorption); multifunctional integration (diagnostic and therapeutic integration)	Lack of standardized preparation; uncertain long-term biosafety; protein corona formation reduces targeting efficiency	[64, 66, 82, 86]
	Nucleic acid aptamer capture	Aptamers screened by SELEX bind targets with high specificity	Small molecular weight, good stability; can be multivalent (e.g., 3DNAwalker for increased sensitivity)	Aptamers are easily degraded by serum nuclease; affinity is affected by the biological environment; long lead time to clinical translation	[91, 96]
	Magnetic nanoparticle technology	Surface-functionalized magnetic particles (antibodies/aptamers) for rapid CTC enrichment in a magnetic field	Easy handling, fast separation; suitable for large samples (e.g., HECMMNs to capture multicancer CTCs)	Strong magnetic field impairs cellular activity; rare subpopulations (CTC clusters) are easily lost; high concentration triggers particle aggregation.	[98–100]

## CTC detection techniques

The development of CTC detection technology provides an important tool for phase diagnosis, prognosis assessment and treatment monitoring of tumors. Based on the key role of CTC in tumor diagnosis and treatment, how to achieve high purity and high recovery rate of CTC isolation and detection has become a core challenge for the clinical translation of liquid biopsy technology. Currently, CTC detection technology mainly focuses on the four key aspects of “capture-enrichment-recognition-analysis”, and continuously improves the detection performance through multidisciplinary cross-fertilization. In the following sections, we will systematically introduce the technical principles, latest progress, and clinical application value of three major categories of CTC detection methods: nucleic acid-based, flow cytometry-based, and optical/nanotechnology-based approaches.

### Nucleic acid-based detection

Nucleic acid-based CTC detection technology enables high sensitivity by identifying tumor-specific gene mutations and expression profiles, demonstrating substantial potential potential for early tumor detection and dynamic monitoring. With the breakthroughs in molecular biology technologies such as single-cell sequencing and digital PCR, the detection of nucleic acid markers has become one of the most promising approaches in CTC research.

#### PCR-based CTC nucleic acid detection

Polymerase chain reaction (PCR) has become an important tool for CTC nucleic acid detection due to its excellent sensitivity, and breast cancer CTCs can be effectively identified by detecting tumor-specific mRNAs, such as hMAM and CK19, in the blood, with a sensitivity of 1 CTC/10^6^ blood cells [105–108]. With the advancement of technology, digital PCR (dPCR) has significantly improved detection accuracy by enabling absolute quantification of nucleic acids. As summarized by Boukouris et al., dPCR has been reported to detect mutant allele frequencies as low as 0.001% in lung cancer patients [109]. While reverse transcription PCR (RT-PCR) is capable of detecting multiple markers simultaneously (e.g., EpCAM, CK19, HER2), its specificity is often limited by abnormal gene expression in hematopoietic cells [108]. To address this limitation, Smilkou et al. utilized droplet digital PCR (ddPCR) to detect ESR1 mutations in gDNA extracted from CTCs, reporting a higher detection rate in CTCs compared to paired plasma cell-free DNA (cfDNA) [110].

#### NGS-based molecular analysis of CTCs

Next-generation sequencing (NGS) and single-cell sequencing technologies are significantly advancing CTC research by characterizing genomic features with unprecedented precision. Innovations in microfluidics have facilitated this progress; for instance, the Hydro-Seq system enables high-throughput single-cell RNA sequencing (scRNA-seq) of CTCs by effectively clearing blood cells and capturing rare tumor cells for transcriptome analysis [111]. Furthermore, single-cell ATAC-seq (scATAC-seq) is emerging as a powerful tool to profile the chromatin accessibility of CTCs. Lohr et al. [112] revealed the mutation profiles and clonal evolutionary trajectories of CTCs by single-cell whole-exome sequencing, which provided an important tool to understand tumor heterogeneity. Ni et al. [113] utilized single-cell whole genome amplification (e.g., MALBAC) to analyze CTCs. They revealed, for the first time at the single-cell level, a highly consistent copy-number variation (CNV) pattern between CTCs and metastatic lesions, identifying CTCs as the origin of metastases. This provides molecular support for the “seed-soil” theory.

At the transcriptome level, Massague and Obenauf [114] found that the WNT signaling pathway was frequently activated in metastatic CTCs using single-cell RNA sequencing, a finding that provides a potential target for the development of anti-metastatic therapy. The technological advances are driving the translation of CTC research from basic exploration towards clinical application.

#### Emerging nucleic acid markers and epigenetic testing

In recent years, CTC nucleic acid marker research has extended beyond the scope of traditional DNA/RNA analysis and expanded to a broader dimension. Taylor et al. [115] demonstrated that exosome microRNAs (miRNAs) derived from CTCs hold diagnostic and therapeutic potential for ovarian cancer. Epigenetic analysis has also opened up new avenues for CTC research. Furthermore, recent progress [116] highlights that integrating multi-omics data with the assessment of CTC survival status provides a promising strategy for defining functional subgroups and deepening our understanding of tumor heterogeneity.

Facing technical bottlenecks, researchers have proposed innovative solutions: Cheng and colleagues [111] developed the Hydro-Seq platform, which utilizes barcoded beads to capture and sequence transcriptomes of CTCs. By effectively eliminating leukocyte contamination, this technology enables high-resolution molecular characterization of CTCs at the single-cell level. Similarly, Guo et al. [117] developed a dual-recognition interface smart response platform based on pollen-mimicking topologically-structured magnetic nanoparticles (IP-GSMNs). This system aims to achieve efficient capture and non-destructive release of CTCs and enhance the sensitivity of CTC detection through epigenetically stabilized surface markers (e.g., EpCAM) and functional nucleic acid modification and specificity. Together, these technological advances are driving the development of CTC nucleic acid analysis in the direction of higher sensitivity, higher throughput, and greater precision.

In summary, CTC detection technology based on nucleic acid analysis has become a pivotal development direction in the field of liquid biopsy. From highly sensitive PCR technology to high-resolution single-cell sequencing, nucleic acid detection methods are continually evolving to address technical bottlenecks. With the development of emerging technologies such as epigenetic analysis and exosome detection, CTC nucleic acid marker detection is rapidly evolving in the direction of multiomics integration, which provides a robust technical support for the improvement of the tumor precision diagnosis and treatment system. However, despite these advances, NGS-based approaches remain cost-intensive and require complex bioinformatic analysis, which currently limits their routine widespread implementation in clinical laboratories.

### Flow cytometry -based detection

Flow cytometry has established itself as a powerful platform in CTC detection due to its high throughput and multi-parameter analysis capabilities. In recent years, with the innovative development of novel fluorescent probes, mass spectrometry labels and detection instruments, flow cytometry has achieved significant improvement in the sensitivity and specificity of CTC detection.

#### Traditional flow cytometry detection

Microfluidic technologies utilizing specific recognition of CTC surface markers have made significant strides in tumor liquid biopsy. Nagrath et al. [118] developed the ‘CTC-Chip’, a microfluidic device containing anti-EpCAM-coated microposts. This technology demonstrated high sensitivity, reportedly detecting 5-1281 CTCs/mL in approximately 99% of patients with advanced cancers in their study cohort. To improve capture efficiency, Stott and colleagues [119] developed the HB-Chip, which incorporates herringbone grooves to generate microvortices. This design enhances the chaotic mixing of blood flow, thereby enhancing the interaction between CTCs and the antibody-coated surface.

However, challenges remain regarding detection limits and purity, particularly for early-stage diagnosis. To address these limitations, Rao et al. [120] developed a biomimetic immunomagnetosome technology coated with leukocyte membranes. This approach aims to reduce non-specific adsorption through biological camouflage, improving the detection signal-to-noise ratio and enabling the sensitive detection of approximately 1 CTC/mL.

#### Image flow cytometry

Image flow cytometry enables efficient detection and characterization of CTCs by combining the high-throughput advantages of flow detection with the morphological analysis capabilities of microscopic imaging. While the flow detection technology is continuously optimized, the integration of microfluidics has introduced novel avenues for CTC detection. Geometrically activated surface interaction (GASI) microarray developed by Hyun et al. [121] adopts the design of biomimetic herring bone structure and optimizes the hydrodynamic properties. This design enhances cell-substrate interactions to improve capture efficiency, providing a promising technological platform for potential clinical CTC detection.

#### Mass cytometry and novel probe technologies

By utilizing metal isotope labels instead of traditional fluorescent dyes, mass Cytometry (CyTOF) technology has broken through the technical bottleneck of spectral overlapping, enabling high-dimensional multi-parameter detection. Bose and colleagues [122] employed a CyTOF-based platform with a 20-marker antibody panel to profile CTCs in small cell lung cancer (SCLC). Their study revealed that specific CTC subpopulations and EMT states are significantly associated with tumor aggressiveness and therapy response, providing a personalized approach to understanding metastasis and resistance mechanisms. Meanwhile, the development of functionalized nanoprobes has further advanced detection performance: Wu et al. [123] designed a gold nanostar surface-enhanced Raman scattering (SERS) probe with a detection sensitivity of 1 CTC/mL, highlighting the potential of this approach for high-sensitivity liquid biopsy.

In conclusion, flow cytometry has evolved from traditional detection to intelligent analysis, serving as a robust platform for CTC characterization. While enabling a new stage of functional state analysis and precision treatment, current flow cytometry approaches still face challenges in preserving nucleic acid quality, as the necessary permeabilization for intracellular staining may limit subsequent molecular profiling.

### Optical and nanotechnology-based detection

Microscopic imaging technology remains the gold standard in the field of CTC detection due to its advantages of direct visualization which ensures high specificity and reliability. With the iterative development of the technology, the field has evolved from the initial traditional optical microscope observation to a modern detection platform integrating advanced technologies such as automated scanning, digital image processing, and artificial intelligence analysis, which has realized a substantial improvement in detection efficiency and accuracy.

#### Nanotechnology-based capture and identification

CTC detection technology has evolved towards higher sensitivity and multiplexing capabilities. Yoon et al. [124] developed a functionalized graphene oxide (GO) chip, which utilizes the nanoscale interface to enhance the capture efficiency of CTCs, improving detection sensitivity even at low concentrations. Building on capture technology, Zheng et al. [125] developed aptamer-functionalized barcode particles. This system allows for the simultaneous capture and specific identification of multiple types of CTCs, providing a powerful tool for analyzing the heterogeneity of circulating tumor cells.

#### Microfluidic chip and microimaging integration technology

The rapid development of microfluidic technology has provided diverse platforms for CTC isolation. Bhagat and colleagues [126] developed a negative selection chip that substantially improves the purity of CTCs by specifically removing CD45-positive leukocytes, creating favorable conditions for subsequent analysis. In terms of positive capture, Cheng et al. [127] employed a 3D scaffold chip integrated with a thermosensitive coating. This design not only achieves high capture efficiency (84%) through specific topographic interactions but also allows for the reversible release of viable CTCs by lowering the temperature.

#### Advanced optical imaging and artificial intelligence analysis

The integration of Artificial Intelligence (AI) with optical imaging is advancing CTC detection. Commercial implementation of these algorithms is already underway to standardize CTC detection. For instance, BioView employs advanced AI-driven imaging software in its Duet™ system, which automates the scanning and classification of cells based on morphological features and fluorescence signal intensity. According to the company’s technical specifications, their AI algorithms are trained to differentiate potential CTCs from hematologic cells by analyzing multi-parametric data, thereby streamlining the manual workload required for review [128, 129]. Similarly, Abnova has integrated this AI-powered imaging technology into its liquid biopsy workflow, combining it with their enrichment platforms to provide an integrated system aimed at automating rare cells and reduces inter-observer variability [130].

In academic research, label-free approaches like digital holographic microscope (DHM) have gained attention. DHM can efficiently differentiate CTCs from blood cells via quantitative phase imaging, offering a distinct advantage in detecting EMT-type CTCs, which might be missed by traditional immunocapture. For example, Gangadhar and colleagues [131] developed a deep learning-assisted DHM system that enables the in-flow enumeration of tumor cells, offering a rapid, stain-free alternative for objective quantification. Conversely, multi-modal strategies have advanced the management of hepatocellular carcinoma (HCC). Several studies [132–134] have utilized antibody cocktail-based in vivo capture combined with specific aptamer fluorescence imaging to identify tumor origin, while further validate biomarkers like PD-L1 to predict the efficacy of combined radiotherapy and antiangiogenic therapies.

However, the field still faces the challenge of standardization, and a study by Alix-Panabieres and Pantel [135] pointed out that there is a 30% difference in CTC morphology interpretation standards between different laboratories. In this regard, the International Liquid Biopsy Consortium (ILBC) is actively promoting the establishment of uniform testing standards, an initiative that is expected to enhance the reproducibility and clinical translational value of CTC testing.

In summary, microscopic imaging technology continues to innovate and develop in the field of CTC detection, gradually evolving from traditional optical microscopy to a modern detection platform that integrates automation, digitization and AI. Currently, the advancement of international standardization will further promote the application of this technology in clinical translation.

## Molecular characterization and biological profiling of CTCs

### Surface markers

Surface markers are commonly employed for the isolation and typing of CTCs, enabling the capture and identification of CTCs through differences in the expression of specific antigens. These markers are usually based on biological characteristics retained by tumor cells during metastasis. Most cancers originate from epithelial cells, and EpCAM and Cytokeratin (CK) are two of the most classical epithelial markers, the expression of which varies among different cancer types. EpCAM is a transmembrane glycoprotein widely expressed on the surface of epithelial-derived tumor cells, which is involved in tumor proliferation and metastasis by mediating intercellular adhesion [136]. Complementarily, CK is a member of the cytoskeletal protein family and plays a key role in maintaining epithelial cell morphology.

However, the limitations of EpCAM-dependent methods are significant, and down-regulation or even absence of EpCAM expression in CTCs undergoing EMT leads to missed detection [137]. In addition, low levels of EpCAM expression in normal epithelial cells and certain leukocyte subpopulations may trigger false positives [138]. The application of single markers is limited by the heterogeneity of CTCs and dynamic phenotypic changes, and a combination of multi-marker strategies is needed to improve detection sensitivity [139, 140]. Recent studies have partially alleviated this limitation by improving antibody affinity or incorporating multiparameter flow cytometry. For example, the EpCAM/vimentin/GPC3 triple-antibody lipid magnetic bead system has been shown to achieve efficient capture in hepatocellular carcinoma samples, with captured CTCs exhibiting high concordance with genetic mutations identified in matched primary tumor foci [136].

CK is usually detected by immunofluorescence staining (e.g., CK8/18/19) in combination with CD45-negative markers to exclude leukocyte interference, but its expression is also affected by EMT, and certain granulocyte subpopulations may nonspecifically bind to CK antibodies.

The introduction of a second exclusion marker reduces the false-positive rate [139]. Notably, the choice of CK isoforms represents a key factor influencing assay specificity: CK7 is highly expressed in breast cancer, whereas CK20 is commonly found in colorectal cancers, and the specificity of the cancer type can be improved by combining multiple antibodies [141].

EMT is a critical step for CTCs to detach from the primary focus and enter the circulation, accompanied by down-regulation of epithelial markers (EpCAM, CK) and up-regulation of mesenchymal markers (Vimentin, Ncadherin). Mesenchymal-type CTCs are more invasive and correlate with metastatic potential and drug resistance [142]. Vimentin, as an intermediate filament protein, and its cell surface form (CSV) antibody in combination with microfluidics can detect EpCAM-negative CTCs, especially in sarcomas and gliomas [143, 144], but the expression of Vimentin in activated fibroblasts and endothelial cells may interfere with the specificity [145]. Combining EpCAM with Vimentin antibody in prostate and pancreatic cancers has been reported to increase CTC capture efficiency compared to a single antibody [140]. Similarly, CD44 (a stem cell marker) in combination with CK identifies a subpopulation of CTCs with a propensity to metastasize [146]. In addition, multi-omics analysis (e.g., single-cell RNA sequencing) has revealed the dynamic expression of EMT-related genes (e.g., TWIST1, ZEB1) in CTCs, providing a preliminary molecular basis for personalized treatment strategies, and high expression of the EMT transcription factor SLUG in breast cancer CTCs was found to be significantly associated with paclitaxel resistance by single-cell transcriptome analysis [147].

There are significant differences in CTC surface markers in different cancer types, most of which are consistent with molecular markers specific to the primary tumor. In prostate cancer, prostate-specific membrane antigen (PSMA) and androgen receptor (AR) variants (e.g., AR-V7) are important markers [148]. In colorectal cancer, high expression of CEACAM6 (carcinoembryonic antigen-associated cell adhesion molecule 6) in cerebrospinal fluid CTCs correlates with leptomeningeal metastasis [149].Besides surface markers, the heterogeneity of CTCs is reflected in phenotypic, genotypic and functional diversity. For example, epithelial (CK+/Vim-), mixed (CK+/Vim+), and mesenchymal (CK-/Vim+) CTCs can coexist in breast cancer, with mixed CTCs being associated with an increased risk of stage 1 recurrence [150]. At the genetic level, single-cell sequencing revealed the spatiotemporal evolution of CTC-driven mutations, and in metastatic prostate cancer, CTCs from the same patient at different time points can present PIK3CA mutations [151]. Functional heterogeneity, on the other hand, is manifested in stem cell properties and drug efflux capacity. For example, CD133 + CTCs in hepatocellular carcinoma highly express the ABCG2 drug transporter protein, the amount of which directly correlates with sorafenib resistance [136]. To resolve this complexity, emerging technologies such as metabolic labeling combined with microfluidic sorting show potential. An assay system based on the metabolic engineering of sialic acids, by labeling CTC surface glycans with exogenous sugar precursors and combining them with anti-fluorescent labeling antibodies to achieve unbiased capture, has successfully isolated a subpopulation of EpCAM-/Vimentin + CTCs in breast cancers, and demonstrated that they have a greater in vivo tumorigenic capacity than epithelial-type CTCs [152].

Therefore, there is spatiotemporal heterogeneity among different cancer types and in the same patient, and it is difficult to define the entire CTCs cohort with a limited number of molecular markers. and current CTC surface marker studies are moving toward high sensitivity and multi-omics integration. For example, in colorectal cancer, the combination of APC gene mutation analysis and CK/Vimentin phenotypic assay combined with gene mutation analysis and phenotypic assay to improve the traceability accuracy [153]. In addition, nanotechnology-driven novel probes (e.g., peptide-functionalized magnetic beads) can simultaneously capture EpCAM low-expressing CTCs and detect their surface receptor activity. In melanoma, researchers identified a class of circulating hybrid cells (hybrids) expressing both immune and tumor proteins, which co-express proteins such as CD74, TMSB10, and GPX1 on their surfaces, and show stronger migration and immune escape ability, providing a new perspective on the phenotyping of CTCs, which not only have a higher detection rate than typical CTCs in peripheral blood, but also show potential for metastasis prediction. Regarding hybrid phenotypes, Gast and colleagues [154].identified neoplastic-immune hybrid cells in uveal melanoma patients. Detected via the co-expression of tumor and immune markers, these hybrid cells demonstrated significantly higher metastatic potential than solitary CTCs, highlighting the clinical value of analyzing tumor-immune fusion events for metastasis prediction. In the future, AI-driven CTC phenotypic classification systems hold promise for addressing current limitations in CTC identification. For example, deep learning models can automatically distinguish true and false positives by analyzing the co-localization patterns of CTC morphology and surface markers, a capability that could enhance diagnostic specificity in lung cancer applications, pending validation in independent clinical cohorts [155]. The combination of liquid biopsy and imaging histology is expected to achieve more metastasis warning [156].

### Gene mutation and copy number variant analysis

The molecular characterization of circulating tumor cells provides insights into understanding tumor heterogeneity, dynamic evolution, and therapeutic resistance mechanisms, and gene mutations and copy number variations (CNVs), as core features of genomic instability, provide an important window for revealing the molecular properties of CTCs. Advances in single-cell sequencing technology have facilitated the analysis of whole genome, exome, and transcriptome of CTCs, especially in revealing the spatial and temporal heterogeneity of driver mutations (e.g., EGFR, KRAS, and TP53) and CNVs [157, 158]. Mutations carried by CTCs may reflect the dynamic evolution of the primary tumors and metastatic foci. For example, high expression of KRAS mutations in CTCs of colorectal cancer (CRC) patients is associated with tumor perforation, lymph node metastasis and distant metastasis [159]. Single-cell exome sequencing technology further revealed the presence of TRPS1 R544Q mutation in CTCs from liver metastases of colon cancer, which enhances tumor aggressiveness through the TRPS1/ZEB1 axis and is associated with advanced disease and poor prognosis [157]. In addition, TP53, EGFR and PIK3CA mutations detected in preoperative CTCs of non-small cell lung cancer (NSCLC) patients were significantly associated with the risk of postoperative recurrence, suggesting that they serve as potential markers for microscopic residual disease (MRD) surveillance [160]. In metastatic bladder cancer, whole transcriptome sequencing of CTC revealed that amplification of chromosome 8q24 (containing the MYC gene) was associated with an invasive phenotype, whereas deletion of chromosome 9p21 (containing CDKN2A) was correlated with chemoresistance [161]. During colorectal adenoma-carcinoma progression, copy number gains on 20q13.2 (AURKA-containing) and 8q24.21 (MYC-containing) have been shown to be stage events of malignant transformation, while consistent with findings in ctDNA or exosomes, CTC-derived CNVs in liquid biopsies provide early warning of metastatic risk [162]. Notably, the mutational spectrum of CTCs may be independent of the primary tumor, for example, in colorectal cancer patients, some CTCs carry KRAS or PIK3CA mutations not found in matched primary tissues, highlighting the uniqueness of the molecular signature of CTCs [158]. In prostate cancer, high-content analysis and single-cell sequencing of CTCs have revealed the dynamic evolution of tumor genomes under therapeutic pressure. Dago et al. [163] identified distinct CTC subpopulations that emerged during treatment, characterized by unique copy number variations (e.g., MYC amplification) and evolving androgen receptor (AR) genotypes. These findings suggest that genomic instability drives the selection of drug-resistant clones, emphasizing the value of CTCs in monitoring the rapid phenotypic and genomic shifts associated with tumor progression.

Innovations in single-cell sequencing technologies have greatly improved the precision of CTC molecular analysis. For example, a mesophoretic-based microtiter system combined with genome-wide amplification technology can detect KRAS, BRAF, and PIK3CA mutations from individual CTCs while resolving the spatial heterogeneity of CNVs [158]. In addition, transcriptome analysis of CTCs can reveal the expression dynamics of immunomodulatory genes (e.g., CTLA-4, PD-L1), whose synergistic effects with KRAS mutations may provide new targets for combination immunotherapy [159]. Multi-omics integration strategies (e.g., combined CTC-DNA and ctDNA analysis) have further improved the sensitivity of mutation detection [164].

Despite the notable advances in CTC molecular analysis, many challenges remain. First, the rarity and heterogeneity of CTCs require the development of highly sensitive capture platforms, such as graphene oxide-based microfluidic chips for simultaneous CTC enrichment and EGFR/HER2 protein expression analysis [161]. Secondly, the molecular differences between CTCs and primary tumors need to be verified by longitudinal tracking for clinical relevance, e.g., the high expression of STAT3/PRKCB in CTCs from breast cancer patients can predict CDK4/6 inhibitor resistance [165]. In the future, AI-driven single-cell multi-omics analysis of CTCs is expected to resolve the tumor evolutionary tree and guide individualized treatment [166].

### mRNA expression profiling (transcriptome) assays

The mRNA expression profiling of CTCs provides a critical perspective for understanding tumor heterogeneity, metastatic mechanisms, and therapeutic resistance by revealing dynamic changes at the transcriptome level. For example, in metastatic breast cancer, CTCs present a mixed epithelialmesenchymal phenotype (EpCAM+/VIM+), in which high expression of stem cell-related genes (e.g., ALDH1, CD44) is significantly correlated with shortened progression-free survival of patients, and such CTCs enhance self-renewal through activation of the Wnt/ β -catenin pathway, and contribute to chemoresistance [167]. Notably, overactivation of the Wnt signaling pathway in prostate cancer CTCs is strongly associated with abiraterone resistance, and their transcriptomic profile may predict the risk of treatment failure [168].

The transcriptomic profile of CTCs can serve as a valuable biomarkers for patient stratification. In mCRPC, high expression of PSMA and PSA mRNA is associated with advanced tumor stage and rapid disease progression [169]. MBNL1-mediated selective splicing events in pancreatic ductal adenocarcinoma (PDAC) CTCs drive metastasis through the regulation of EMT-related isoforms (e.g., CD44v6), and their splicing patterns reflect tumor microenvironmental stress [170]. Transcriptome dynamics of metastatic CRC CTCs revealed elevated expression of the epigenetic regulators EZH2 and HDAC6 during disease progression, which may be potential targets for combined deacetylase inhibitors [171].

Despite significant technological advances, transcriptome analysis of CTCs is still limited by rare cell capture and low starting RNA amounts. Novel microfluidic platforms (e.g., Parsortix^®^) improve CTC recovery through label-free enrichment strategies and enable highly sensitive detection in combination with targeted Panel sequencing [172]. Artificial intelligence-driven image analysis techniques identify subpopulations of breast cancer CTCs (e.g., SLR-type), whose morphology-transcriptome association features (high mitochondrial density, rough membranes) correlate with stem cell characteristics and poor prognosis [173].

Furthermore, Tumor-Educated Platelets (TEPs) represent an emerging biomarker source in liquid biopsy. TEPs carry tumor-derived RNAs that are protected by platelet membranes from degradation by circulating RNases, enabling non-invasive molecular tumor typing and dynamic monitoring. Although distinct from CTC surface marker detection, the molecular characterization of TEPs serves as a valuable complementary assay. It offers potential for the real-time assessment of tumor molecular profiles and indirectly contributes to the improvement of CTC detection strategies [174].

### Proteomic analysis

Proteomic analysis of CTCs aims to systematically resolve the expression profiles of their surface and intracellular proteins and reveal molecular features related to metastasis, drug resistance and immune escape. In contrast to genomic and transcriptomic profiles, proteomic data more directly reflects the complement of functionally active molecules, especially post-translational modifications (e.g., phosphorylation) and protein-interaction networks, and provides a dynamic perspective for understanding the biological behavior of CTCs [175]. Despite the low abundance of single-cell proteins and sample scarcity, the combination of mass spectrometry, immunofluorescent labeling, and microfluidic platforms has significantly advanced the development of proteomics of CTCs in recent years. Mass spectrometry-based proteomics improves targeting by detecting thousands of proteins in CTCs with high sensitivity, combined with immunoenrichment or size-filtering techniques. For example, the ZeptoCTC workflow, which combines single-cell isolation with reversed-phase protein microarrays, enables precise quantification of Akt and Erk phosphorylation levels in CTCs. It was found that CTCs from patients with metastatic breast cancer had significantly higher pAkt/pErk ratios than matched leukocytes, suggesting that the activity of the PI3K/AKT/mTOR pathway is correlated with metastatic potential [176]. Single-cell mutation profiling of melanoma CTCs by mass spectrometry revealed ctDNA-undetected driver mutations (e.g., NRAS Q61K), highlighting the complementary nature of CTC proteomic and genomic analyses [177]. In addition, immunofluorescence multicolor labeling allows for simultaneous analysis of surface markers and intracellular signaling proteins in CTCs. Capture of CDCP1-expressing CTCs by magnetic activated sorting (MACS) was found to correlate with the mesenchymal phenotype and chemoresistance of triple-negative breast cancer, providing a new direction for targeted therapy [178].

The expression of immune checkpoint molecules (e.g., PD-L1, CTLA-4) in CTCs is a current research hotspot. In locally advanced breast cancer, it was found that there was no significant change in the PD-L1 expression of CTCs before and after radiotherapy, but baseline PD-L1-positive patients were more likely to show progression as defined by the RECIST criteria, underscores their potential prognostic value [179]. Phosphorylated protein analysis can reflect the signaling pathway activity of CTCs in real time. Metabolomics combined with phosphorylated protein assay revealed that the “one-carbon metabolism” pathway (e.g., the folate cycle) was abnormally activated in liver metastases originating from CTCs, and the mRNA and protein expression of related enzymes (e.g., MTHFD1) were upregulated in synchrony, suggesting that the metabolic reprogramming was a synergistic mechanism with metastasis [180].

However, standardization of the technique remains a bottleneck. Comparison of the proteome of cerebrospinal fluid CTCs with cell-free DNA (cfDNA) revealed low concordance in copy number variation (CNA), suggesting that single-cell heterogeneity may affect the interpretation of results [181]. Integration of multi-omics (protein + metabolism + genome) and artificial intelligence will further enhance the resolution of the CTC proteome. The development of the " dSCOUT” platform based on single-cell metabolic fingerprints (e.g., ASGPR, GPC-3) demonstrates the potential of multimodal analyses by mapping the protein-mRNA two-dimensional profiles of CTCs to metastatic risk models through machine learning to further enhance the resolution [182]. Emerging multimodal imaging technologies, such as STAMP (Single Cell Transcriptome and Multimodal Protein Imaging), can realize simultaneous detection of RNA and protein markers in the same cell while preserving cellular morphology and spatial structure, which significantly improves the resolution of CTC heterogeneity, and the technology does not require single-cell sequencing, has low cost and high throughput, and is particularly suitable for the functionality of rare cell populations such as CTCs protein validation and molecular typing, providing a powerful tool for clinical translation [183]. Table 2 summarizes the key molecular markers in CTC typing, their detection methods and clinical significance.

### CTC-centered integrative analysis: microenvironmental interactions and functional implications

While the molecular characterization of isolated CTCs provides critical insights into tumor biology, current research is increasingly shifting towards a holistic approach that integrates multiple liquid biopsy markers and explores the interaction between CTCs and their microenvironment. This “multi-liquid biopsy” strategy can provide complementary information to that derived from single biomarker. For instance, Smilkou et al. demonstrated the clinical value of co-analyzing cfDNA and CTCs, utilizing ddPCR to detect ESR1 mutations and providing complementary genomic information that enhances predictive accuracy [110]. Similarly, to capture a broader spectrum of tumor signals, Kang et al. introduced a dual-isolation platform integrating a microfluidic chip with a hydrogel interface. This system enables the simultaneous capture and profiling of both CTCs and exosomes, offering a more comprehensive view of the tumor burden for cancer diagnosis [184, 185].

Beyond multi-marker integration, the interplay between CTCs and the hematic microenvironment plays a pivotal role in metastasis and immune evasion. CTCs do not travel alone; they actively interact with blood components. Jiang and colleagues discovered that a significant proportion of CTCs in the bloodstream are cloaked by platelets, which shields them from shear stress and immune surveillance. To address this, they devised a specific microfluidic strategy to isolate these platelet-covered CTCs, revealing mechanisms of survival in circulation [186]. Furthermore, the dynamic interaction between CTCs and immune cells has become a focal point of investigation. Using a novel microfluidic system, Kang and Niu et al. observed a negative correlation between the number of CTCs and Natural Killer (NK) cells, suggesting that NK cells are actively involved in clearing CTCs from the circulation [187]. These advances underscore the importance of analyzing CTCs not as isolated entities but as part of a complex, interactive ecosystem.

**Table 2:** Molecular markers for CTC typing: assay modalities and clinical relevance

Markers	Marker class	Detection methods	Clinical implications	References
EpCAM	Epithelial surface protein	Immunomagnetic bead enrichment (CellSearch System); Immunofluorescence staining	Used to enrich epithelial-derived CTCs, but expression is downregulated during EMT and misses mesenchymal-type CTCs. Commonly used in breast, prostate, and colorectal cancers, but sensitivity is affected by tumor heterogeneity.	[103, 137]
CK8/18/19	Cytokeratin (epithelial marker)	Immunofluorescence (CK+/CD45-); RT-PCR to detect mRNA	Distinguish CTC from leukocytes, but some granulocytes may bind non-specifically. CK7 (breast cancer) and CK20 (colorectal cancer) subtype selection improves cancer species specificity.	[139, 141, 188]
Vimentin	Mesenchymal markers	CSV antibody (surface form); Microfluidic chip	Recognizes EMT-type CTCs, more aggressive, correlate with metastatic potential. Superior to EpCAM in sarcomas, gliomas, but activated fibroblasts may interfere with specificity.	[142, 145]
CD44	Stem cell marker	Flow cytometry (CD44+/CK+); Single-cell sequencing	Enrichment of CTC subpopulations with stem cell properties correlates with drug resistance and metastasis. High expression of ABCG2 drug efflux protein in breast and hepatocellular carcinoma suggests chemotherapy resistance.	[152, 189]
AR-V7	Androgen receptor splice variant	EPIC platform (RT-PCR); Single-cell sequencing	Predict resistance to androgen receptor inhibitors (e.g., enzalutamide) in desmoplasia-resistant prostate cancer (mCRPC). AR-V7 + patients may be more sensitive to paclitaxel.	[148, 169, 190]
PD-L1	Immune checkpoint protein	Immunofluorescence (CTC surface); CyTOF	Assesses response to immunotherapy (e.g., anti-PD-1); positive CTCs positively correlate with efficacy. Dynamic monitoring may detect treatment failure earlier than imaging.	[179, 191, 192]
KRAS mutations	Driver gene mutations	ddPCR (low frequency mutations); Single-cell whole-exome sequencing	KRAS mutation CTCs in colorectal cancer are inconsistent with the primary focus, suggesting clonal evolution. Correlates with CTLA-4 expression and may influence immunotherapy strategies.	[159, 162]
PSMA	Prostate-specific membrane antigen	Immunofluorescence (PSMA+); Transcriptome analysis (PSMA mRNA)	Prostate cancer-specific marker, combined with AR-V7 testing to optimize treatment choices. High baseline PSMA expression is associated with rapid progression.	[148, 169]
CEACAM6	Carcinoembryonic antigen-associated adhesion molecule	Microfluidic chip (cerebrospinal fluid CTC); Immunohistochemistry	Highly expressed in cerebrospinal fluid CTC in patients with colorectal cancer soft meningeal metastases and can be used as a prognostic marker. More microenvironment-specific than blood-derived CTC.	[149, 153]
TWIST1/ZEB1	EMT transcription factor	Single-cell RNA sequencing; Multiplex fluorescence in situ hybridization (FISH)	EMT dynamic monitoring: upregulation of TWIST1 is associated with paclitaxel resistance (breast cancer). Mixed CTC (epithelial + mesenchymal) suggests higher risk of recurrence.	[147, 193]
MUC1/MUC16	Mucin	Aptamer capture (microfluidics); Mass spectrometry analysis	Highly expressed in CTC of ovarian and pancreatic cancer, associated with peritoneal metastasis. Can be used as a therapeutic target (e.g. antibody-drug coupler).	[171, 194]

## Clinical applications of CTC

**Fig. 3:**
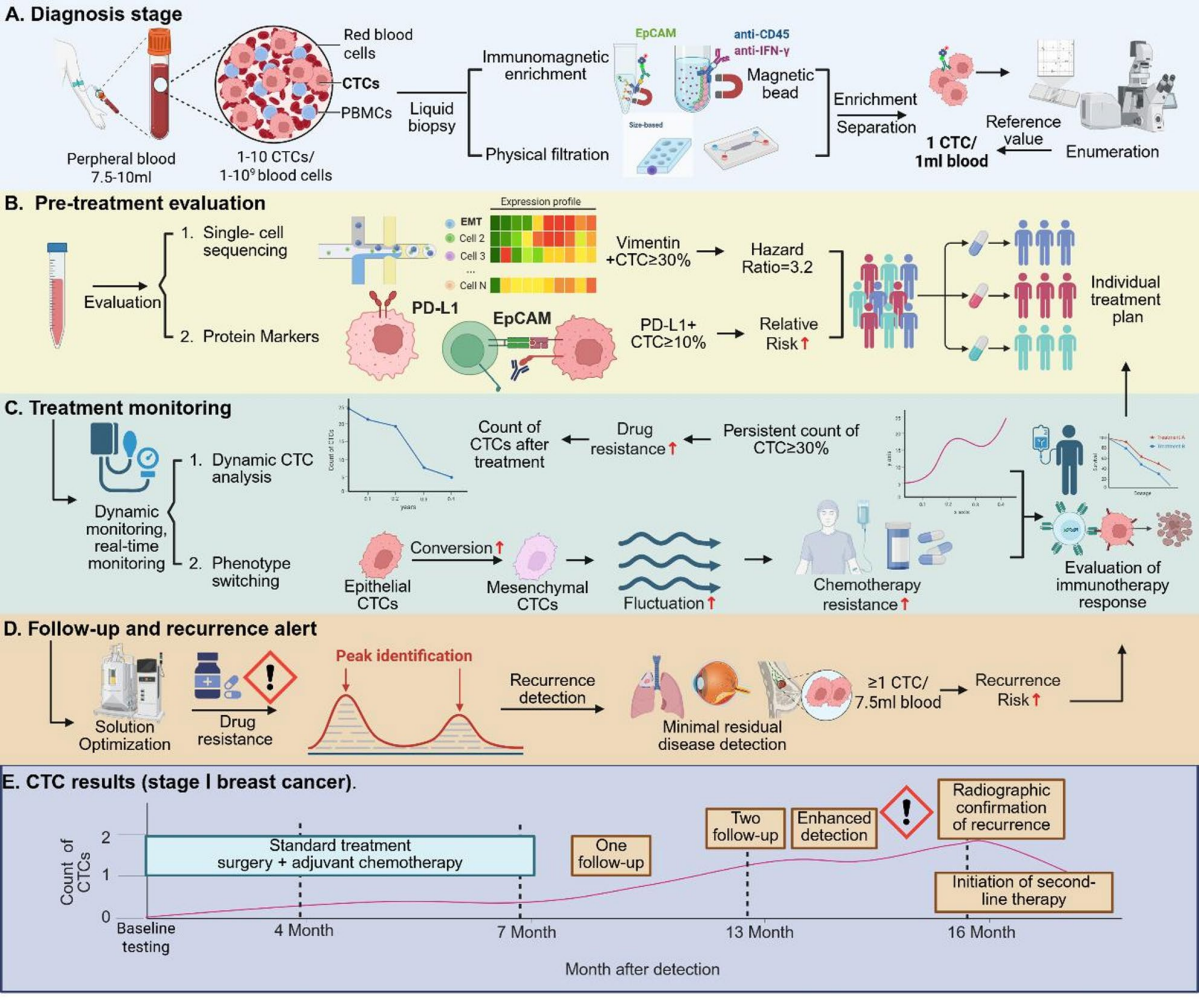
Application of CTCs analysis in clinical decision-making. (**A**) Diagnosis stage: CTCs are isolated from peripheral blood (7.5–10 mL) using physical filtration or immunomagnetic enrichment, enabling sensitive detection (~ 1–10 CTCs/mL). (**B**) Pre-treatment evaluation: Molecular profiling of CTCs via single-cell sequencing and protein marker detection (e.g., EpCAM, PD-L1) helps assess tumor burden, predict therapy response, and guide individualized treatment plans. (**C**) Treatment monitoring: Dynamic CTCs enumeration and phenotype analysis allow real-time evaluation of therapeutic efficacy, early detection of drug resistance, and immunotherapy response assessment. (**D**) Follow-up and recurrence alert: Long-term monitoring of minimal residual disease and phenotypic CTCs switching provides early warning of recurrence and resistance. (**E**) Clinical example: Serial CTCs measurement in a stage I breast cancer patient demonstrates treatment response, recurrence risk, and personalized management value

As an important component of liquid biopsy, CTC have gradually moved from laboratory research to clinical application. In the actual diagnosis and treatment process, CTC analysis not only has the advantages of noninvasive and repeatable sampling, but also can dynamically reflect the evolutionary characteristics of tumors. In recent years, CTCs have shown a wide range of applications in stage screening, recurrence monitoring, efficacy assessment and treatment guidance for various cancers. The following is a systematic introduction of the key value of CTCs in the clinical setting from different application scenarios. Figure 3 summarizes the application of CTCs in clinical diagnosis and treatment, from diagnosis, target discovery to efficacy evaluation.

### Application of CTCs in the diagnosis of early-stage cancer

Compared with the invasive operation and sampling bias of traditional tissue biopsy, CTC detection can obtain tumor information through simple peripheral blood collection. This approach offers significant advantages and holds great clinical value for optimizing patient management and potentially improving clinical outcomes.

#### Diagnostic sensitivity and specificity of CTC detection technology

Current CTC detection technologies can be divided into two categories: marker-dependent (epithelial markers such as EpCAM, CK, etc.) and non-marker-dependent (physical property-dependent). Among them, CellSearch system, as the first FDA-approved CTC detection platform, has established proven value in the prognostic evaluation and monitoring of metastatic breast, prostate and colorectal cancers.

However, this system has inherent limitations due to its reliance on EpCAM antibody capture, which results in a detection rate of less than 30% of CTC subpopulations undergoing EMT [195]. In contrast, the ISET^®^ (Isolation by Size of Tumor Cells) assay system, based on the physical properties of the cells, utilizes 8 μm pore size microporous filtration technology and achieved 80% CTC detection in patients with early-stage NSCLC, demonstrating a significant advantage over the CellSearch system [72].

#### Clinical evidence for CTCs in the diagnosis of early-stage cancers

In breast cancer diagnosis, a study analyzing detection rates in newly diagnosed patients demonstrated a significant positive correlation between the detection rate of CTC and clinical stage [188]. Notably, CTC-positive patients had an increased risk of recurrence at 4 years compared with negative patients, suggesting that CTCs hold potential as a biomarker for indicating the presence of micrometastases.

Pancreatic cancer remains a major clinical challenge to diagnose at early-stage due to its deep anatomical location. The MetaCell^®^ system successfully detected CTC in the peripheral blood of patients with resectable pancreatic cancer by cell-size-based enrichment in combination with short-term in vitro culture, and its detection rate was independent of the metastatic status of the tumor [196]. This important finding suggests that CTC detection technology offers a complementary approach to potentially address some limitations of traditional imaging and open up new avenues for the early diagnosis and accurate staging of pancreatic cancer.

#### Multi-parameter combined detection strategy

Traditional single-indicator CTC detection methods often face limitations in sensitivity and specificity for clinical application. Consequently, new strategies based on multi-parameter integration are becoming a area of intense investigation. However, the effective implementation of such comprehensive analyses is often hindered by two key challenges: the extreme rarity of CTCs and their high phenotypic heterogeneity. To address these technical hurdles, Lapin et al. [197] developed the MINDEC negative enrichment technology. This method specifically depletes CD45 + leukocytes while preserving biologically active CTCs, achieving a mean recovery rate of 82% with effective leukocyte depletion (residual WBCs ~ 400 cells/mL). Preserving viable CTCs with such efficiency provides a vital foundation for downstream applications, as it provides ideal material for single-cell sequencing and proteomic profiling, thereby enabling a comprehensive multi-dimensional characterization of the tumor.

### Application of CTC in prognostic assessment and monitoring of treatment efficacy

The number and molecular characteristics of CTCs are closely related to the prognosis of cancer patients, making them ideal biomarkers for real-time monitoring of treatment response. Unlike traditional imaging and serum protein markers which often lag in reflecting tumor burden, CTC assays can provide a more dynamic and timely assessment of tumor biological behavior.

#### CTC counting and prognostic assessment

A large pooled analysis of individual patient data from 1,944 metastatic breast cancer patients confirmed the independent prognostic value of CTC enumeration. Patients with baseline CTC counts ≥ 5/7.5 mL (46.9%) exhibited significantly reduced progression-free survival (PFS) (HR 1.92, 95% CI 1.73–2.14, *p* < 0.0001) and overall survival (OS) (HR 2.78, 95% CI 2.42–3.19, *p* < 0.0001) compared to those with < 5 CTCs [198]. This prognostic relevance was also validated in mCRPC, where the median OS was significantly shorter by 10.7 months in patients with a baseline CTC count of ≥ 5 compared to those with favorable counts (< 5 CTCs) [199], further highlighting the important role of CTC counts in the prognostic assessment of multiple malignancies.

In addition, the prognostic value of CTCs has broader clinical significance, extending beyond advanced tumors to early-stage cancers. A prospective study by Janni et al. [200] found that the disease-free survival (DFS) of patients with detectable preoperative CTCs (≥ 1/7.5 mL) was significantly reduced compared with CTC-negative patients. This prognostic relevance was also validated in digestive tract tumors. Similarly, the enumeration of CTC based on cellular physical properties can predict recurrence in patients undergoing surgery for early-stage hepatocellular carcinoma (HCC) [201]. Additionally, as biomarkers, CTCs provide valuable prognostic information for patients with early-stage pancreatic cancer [202]. Collectively, these findings further confirm the clinical value of CTC detection in the prognostic evaluation of early-stage tumors.

#### Dynamic monitoring of treatment response

Existing studies have demonstrated that CTC assay has distinct advantages over traditional imaging in treatment monitoring, enabling near real-time assessment of efficacy. Khan et al. [203] demonstrated from a cohort of 138 patients with metastatic neuroendocrine tumors that changes in the number of CTCs after 15 weeks of treatment possessed superior predictive value for prognosis compared with Chromogranin A (CgA).

Although the SWOG S0500 clinical trial failed to demonstrate that CTC count-based adjustments to the phase 1 treatment regimen improved the overall survival of patients with metastatic breast cancer, its subgroup analysis suggested that patients with specific molecular subtypes might benefit from it [204]. This finding suggests that future clinical utility will likely depend on molecular characterization rather than enumeration alone to guide specific targeted therapies.

#### CTC molecular characterization and precise monitoring

In recent years, with the breakthroughs in single-cell analysis technology, molecular characterization of CTCs has opened up a new way for precise efficacy prediction. Scher et al. [190] used the EPIC platform to detect AR-V7 splice variants in CTCs from patients with mCRPC. They found that AR-V7-positive patients had poor response to ARSI treatment, but still exhibited significant sensitivity to taxanes. Parallel advancements have been made in small cell lung cancer (SCLC); for instance, Carter et al. [205] successfully identified significant copy number variation differences between chemotherapy-sensitive and drug-resistant groups by whole genome sequencing of CTCs from SCLC patients. These studies provide an important molecular basis for the selection of second-line clinical treatment.

Meanwhile, the new generation of single-cell protein analysis technologies, represented by single-cell protein blotting (scWB) and microfluidic microarrays (e.g., SAIF, AFM, i.e., SCI, and Orcs-proteomics) [206], have realized the high-efficiency isolation of CTCs and multi-parameter protein expression analysis. These platforms enable the quantitative detection of hormone receptor subtypes (e.g., ER-α 46/ER-α 66) and signaling pathway proteins (e.g., pAKT, pS6) at the single-cell level, providing high-resolution molecular phenotypic information for prognostic assessment, therapeutic monitoring, and drug resistance analysis in breast cancer.

In summary, CTCs assays have demonstrated significant value in tumor therapy, both as an independent prognostic indicator and for real-time monitoring of therapeutic efficacy, with the number of CTCs significantly correlating with patient survival and their molecular characterization predicting drug sensitivity. Compared with traditional methods, CTC assay offer a more dynamic reflection of treatment response, thereby guiding clinical decision-making. With the development of single-cell analysis technology, CTC detection is promoting the development of tumor therapy in the direction of precision and individualization.

### Application of CTC in drug resistance mechanism research and therapeutic target discovery

As a “real-time biopsy” sample of primary tumors and metastases, CTC provides a unique window to study the mechanisms of cancer drug resistance and discover new therapeutic targets. Compared with traditional tissue biopsies, CTC analysis is able to capture spatial and temporal tumor heterogeneity and monitor dynamic evolution.

#### CTC reveals drug resistance mechanisms

The phenotypic plasticity of CTCs plays a key role in tumor progression and drug resistance. Yu et al. [193] found that the expression levels of EMT markers (e.g., Vimentin) exhibited dynamic shifts throughout the course of treatment by longitudinal dynamics of CTCs in breast cancer, and that such phenotypic alterations were closely associated with the development of clinical drug resistance. Single-cell RNA sequencing further identified subpopulations of heterogeneously phenotyped cells expressing both epithelial and mesenchymal markers in CTC populations, which may have enhanced metastatic potential and survival advantages [207].

In mCRPC, CTC molecular analyses revealed multiple mechanisms of resistance, including alterations in AR signaling pathways (e.g., AR-V7 splice variants, AR gene amplification), neuroendocrine differentiation, and dysregulation of ribosome biogenesis [208]. Similarly, single CTC whole-exome sequencing analysis based on the DEPArray™ system showed that chemoresistance in small cell lung cancer was significantly associated with MYC family gene amplification, which provided new molecular insights into the mechanisms of tumor drug resistance [209].

#### CTC guides individualized therapy

CTCs have demonstrated promising clinical value in drug sensitivity prediction. The Collagen Adhesion Matrix (CAM) functional assay platform developed by Pearl et al. [194] revealed significant differences in the sensitivity of invasive CTCs (iCTC) to platinum drugs in ovarian cancer patients, providing experimental evidence to guide individualized drug use. In the field of HER2-positive breast cancer, Giordano et al. [210] successfully predicted the onset of secondary resistance to anti-HER2 therapy by dynamically monitoring the changes in HER2 expression level of CTCs.

In genomics research, De Luca et al. [211] sequenced the whole genome of breast cancer CTCs and found that there was a significant correlation between mutations in the PI3K/AKT pathway and the sensitivity of mTOR inhibitors, which provided a new idea for targeted therapy screening. More notably, the PDX model based on CTC in vitro culture has become valuable platforms for preclinical drug screening, which facilitates the research and development of new anti-tumor drugs.

#### Research on CTC in immunotherapy

Studies on the immune escape mechanism of CTC have provided important insights into cancer immunotherapy. Currently, PD-L1 expression on the surface of CTCs has become a key biomarker for predicting response to immunotherapy. Heymann et al. [192] demonstrated a significant positive correlation between the percentage of PD-L1 positivity in CTCs and the response to anti-PD-1 therapy. Through an innovative co-culture model of CTCs and autologous immune cells, this study further revealed the critical role of immune checkpoints such as CTLA-4 and TIM-3 in mediating immune escape.

Notably, dynamic monitoring of CTC counts was able to predict immunotherapy efficacy earlier than conventional imaging assessments. Wang et al. [212] confirmed in patients with advanced lung cancer receiving immunotherapy that baseline CTC counts and their dynamic variations during treatment hold significant prognostic value, serving as reliable biomarkers for evaluating therapeutic response. Furthermore, Purcell et al. [213] investigated patients with stage III NSCLC undergoing chemoradiation combined with immunotherapy and found that the presence and dynamic changes of CTCs function as early predictors of disease progression. These indicators can identify high-risk patients with poor prognosis prior to radiographic detection, providing a critical basis for the formulation of personalized clinical treatment strategies.

In conclusion, CTCs provide a new way of “liquid biopsy” for the study of tumor drug resistance mechanisms and the discovery of therapeutic targets. Through single-cell analysis, researchers have revealed the roles of CTC phenotypic plasticity (e.g., EMT dynamics) and key signaling pathways (e.g., AR mutations, PI3K/AKT mutations) in drug resistance, and the CTC functional assay platform can assess differences in drug sensitivity and guide individualized therapy. In addition, PD-L1 expression and immune checkpoint analysis of CTC provide a new basis for immunotherapy response prediction. These findings highlight the unique value of CTC in overcoming drug resistance and developing new therapies. Table 3 organizes the clinical application patterns and key findings of CTC assays in different cancer types.

Despite these promising findings, it is important to acknowledge that the routine clinical implementation of CTC analysis has so far been largely confined to prognostic stratification in metastatic breast, prostate, and colorectal cancers. Although CTCs have been investigated in other malignancies, including lung, ovarian, pancreatic, and hepatocellular cancers, their use in these settings remains largely investigational and has not yet been incorporated into major clinical practice guidelines. Challenges include the lack of standardized assays across different platforms, inter-patient heterogeneity, and the need for large-scale prospective interventional trials to prove that CTC-guided treatment decisions definitively improve patient outcomes compared to standard care [135, 204].

**Table 3:** Clinical applications of CTCs across different oncological contexts

Cancer type	Detection method	Major findings	Clinical results	References
Metastatic breast cancer	CellSearch (EpCAM+); Single-cell RNA sequencing (EMT gene)	Median OS was significantly shorter in patients with CTC ≥ 5/7.5mL (15.3 vs. 26.9 months). EMT mixed CTCs (EpCAM+/VIM+) were associated with early recurrence.	Independent prognostic indicators, the SWOG S0500 trial suggested that dynamic monitoring of CTC could guide chemotherapy adjustment. HER2 loss in CTC of HER2 + patients predicts targeted therapy resistance.	[198, 200, 204, 210]
Non-small cell lung cancer	ISET (size filtration); PD-L1 immunofluorescence	CTC detection rate in early-stage patients is 80% (ISET) vs. 23% (CellSearch). PD-L1 + CTC with an immunotherapy response rate of 78% vs. 12% (negative group).	Non-invasive early diagnosis (screening for high-risk individuals). PD-L1 dynamic monitoring is superior to tissue biopsy and avoids repeat puncture.	[72, 191, 193]
Metastatic prostate cancer	EPIC (AR-V7 mRNA); CTC-iChip (whole genome sequencing)	AR-V7 + patients are resistant to abiraterone but effective on paclitaxel. PIK3CA mutations in CTC correlate with mTOR inhibitor sensitivity.	Avoidance of ineffective therapy (drug cost savings); clonal evolutionary analysis guides combination targeted therapy.	[78, 199]
Colorectal cancer	Microfluidic multi-targeting (EpCAM/CEACAM6); KRAS ddPCR	KRAS mutant CTCs were independent of the primary site, suggesting heterogeneity. TRPS1 mutations in liver metastasis CTCs enhance invasiveness.	Liquid biopsy replaces tissue testing to monitor targeted therapy resistance. CEACAM6 + CTC warns of leptomeningeal metastasis.	[159, 162]
Pancreatic ductal adenocarcinoma	MetaCell^®^ (size + short-term culture); MBNL1 splicing analysis	The CTC detection rate in resectable patients was 66.7%, independent of imaging; MBNL1-mediated CD44v6 splicing drives metastasis.	Early diagnostic value (CA19-9-negative patients supplemented). Splicing isoforms as new drug targets.	[170, 196]
Ovarian cancer	CAM function assay (iCTC); MUC16 mass spectrometry	iCTC showed an 8.7-fold difference in sensitivity to platinum. Patients with abdominal metastases have high CTC expression of MUC16.	Individualized chemotherapy regimen selection. Abdominal lavage fluid CTC is more predictive than blood.	[194]
Small cell lung cancer	DEPArray™ Single cell sequencing; MYC FISH	MYC-amplified CTC correlates with chemotherapy resistance. Notch pathway inactivation promotes clonal escape.	Screening of sensitive populations prior to second-line therapy (topotecan vs. immunotherapy).	[205]
Hepatocellular carcinoma	Triple antibody magnetic beads (EpCAM/Vimentin/GPC3); CD133/ABCG2 flow-through	High concordance of GPC3 + CTC with primary focus mutations. High expression of ABCG2 by CD133 + CTC suggests sorafenib resistance.	Targeted therapy resistance monitoring. Postoperative CTC count predicts recurrence.	[136]
Bladder cancer	Graphene oxide microfluidics (EGFR/HER2); Chromatin open analysis	20q13.2 (AURKA) copy number gain correlates with metastasis. High PD-L1 + CTC response rate to immune checkpoint inhibitors.	Early warning of recurrence in non-muscle invasive bladder cancer. Immunotherapy biomarker exploration.	[161]

## Conclusion and outlook

As an important carrier of tumor heterogeneity and metastatic potential, the continuous advancement of CTCs isolation, typing and functional assessment technologies has opened up new pathways for precision tumor diagnosis and treatment. In this paper, we systematically review the current strategies for CTC isolation, including immunocapture methods based on surface markers, enrichment methods based on physical properties, microfluidic microarray platforms, and integrated multimodal technologies, and summarize the research progress of CTCs in the typing dimension, such as multilevel analysis based on phenotypes, genomes, transcriptomes, and proteomes. Meanwhile, this paper emphasizes the unique value of functional assessment in revealing the biological properties of CTCs and predicting tumor evolution, covering techniques such as single-cell culture, drug resistance detection, and metastatic ability testing.

Despite the remarkable progress in CTC research, there are still many challenges that need to be addressed. Primarily, separation efficiency and sensitivity are still bottlenecks in the development of the technology. Traditional isolation methods relying on epithelial markers such as EpCAM tend to miss mesenchymal CTCs in the EMT state, while isolation methods based solely on physical properties also suffer from insufficient specificity in heterogeneous samples. Furthermore, current CTC detection methods are still mainly focused on patients with advanced disease, and further purification and standardization of the technology are needed to apply it to early-stage diagnosis and micrometastasis identification. Additionally, the phenotypic and genetic characteristics of CTC often change dynamically due to the course of disease, treatment and individual differences, so establishing a unified and dynamic classification standard is the basis for promoting its clinical application.

In terms of clinical application, CTC has great potential in the multidimensional management of cancer. First, before treatment, CTC can be used as a noninvasive risk stratification tool to assist in prognostic stratification and staging judgment; during treatment, CTC can dynamically monitor changes in therapeutic efficacy and assess the sensitivity of chemotherapy or immunotherapy; in the follow-up stage, changes in the number or characteristics of CTC can also be used as an early warning indicator of tumor recurrence and metastasis. Especially in some patients who are difficult to obtain tissue biopsy samples, CTC analysis provides a complementary and alternative source of information. In addition, the combination of clinical decision support system (CDSS) and artificial intelligence algorithms for CTC analysis is expected to promote the optimization and precision of individualized treatment plans.

Moving forward, research could explore the creation of new CTC separation systems with improved throughput, sensitivity, and reduced background noise. Potential approaches might include the use of SERS, droplet microfluidics, programmable nanomaterials, and other cutting-edge technologies to achieve full phenotypic spectrum enrichment of CTCs. Future strategies should integrate CTCs with other liquid biopsy markers such as ctDNA, exosomes and circulating RNA to construct a multi-dimensional “tumor fingerprint” and improve the accuracy of tumor typing and dynamic monitoring. Moreover, it is crucial to expand the depth and breadth of CTC function evaluation, such as single-cell spatial genomics, CRISPR screening and in vivo modeling, to deeply analyze the origin of its heterogeneity and metastatic mechanism. Finally, the establishment of standardized protocols, database and clinical evaluation system, is essential to provide a data basis and evaluation standard for large-scale prospective clinical studies.

In summary, as an important bridge between tumor biology research and clinical translation, the separation, typing and functional analysis technology of CTC is gradually moving towards the direction of precision, automation and individualization. With ongoing progress in interdisciplinary research and technological innovation, CTC is expected to emerge as a key marker across the full spectrum of cancer care—potentially spanning early detection, diagnosis, monitoring, and treatment—ultimately contributing more to the field of precision oncology.

## Data Availability

No datasets were generated or analysed during the current study.

## Abbreviations

CTCs: Circulating tumor cells
FDA: Food and Drug Administration
EMT: Epithelial-mesenchymal transition
DEP: Dielectrophoresis
nDEP: negative dielectrophoresis
pDEP: positive dielectrophoresis
MOFs: Metal-organic skeletons
MNPs-TA: Tannic acid-functionalized magnetic nanoparticles
MET: Mesenchymal-epithelial transition
NGF: Next-generation flow cytometry
AFM Chip: Antibody-functionalized microfluidic chip
ICP-MS: Inductively coupled plasma mass spectrometry
DPPB: DNAzyme-functionalized proteins biotinylated strategy
TDFs: Tetrahedral DNA frameworks
IP-CMNs: Protein corona pre-coating
HE-CM-MNs: Hybrid engineered cell membrane-camouflaged magnetic nanoparticles
PCR: Polymerase chain reaction
dPCR: digital PCR
RT-PCR: Reverse transcription PCR
NGS: Next-generation sequencing
CNV: Copy-number variation
GASI: Geometrically activated surface interaction
SERS: Surface-enhanced Raman scattering
DHM: Digital holographic microscope
ILBC: The International Liquid Biopsy Consortium
CK: Cytokeratin
PSMA: Prostate-specific membrane antigen
AR: Androgen receptor
CRC: Colorectal cancer
NSCLC: Non-small cell lung cancer
MRD: Microscopic residual disease
PDX: Patient-derived xenograft tumor
mCRPC: metastatic Castration-Resistant Prostate Cancer
PDAC: Pancreatic ductal adenocarcinoma
TEPs: Tumor-educated platelets
MACS: Magnetic activated sorting
cfDNA: cell-free DNA
CNA: Copy number variation
OS: Overall survival
DFS: Disease-free survival
CgA: CHROMOGRANIN A
SCLC: Small cell lung cancer
scWB: single-cell protein blotting
CAM: Collagen Adhesion Matrix
iCTC: invasive CTCs
ORR: Objective remission rate
CDSS: Clinical decision support system
